# Supplementary material for: Chemically modified CRISPR-Cas9 enables targeting of individual G-quadruplex and i-motif structures, revealing ligand-dependent transcriptional perturbation
Source: Nat Commun. 2025 Dec 9;17:385. doi: 10.1038/s41467-025-67074-z (PMC12796454; doi:10.1038/s41467-025-67074-z)
Supplement: Supplementary file 1 — Supplementary Information [file 41467_2025_67074_MOESM1_ESM.pdf]

Chemically modified CRISPR-Cas9 enables targeting of individual G-quadruplex and i-motif structures, revealing ligand-dependent transcriptional perturbation

Sabrina Pia Nuccio[1,2], Enrico Cadoni[1,3], Roxani Nikoloudaki[1], Silvia Galli[1], An-Jie Ler[1], Claudia Sanchez-Cabanillas,[1,4] Thomas E. Maher [1,2], Ella Fan[1], Dilek Guneri[5], Gem Flint[1], Minghui Zhu[1], Ling Sum Liu[1], Christopher R. Fullenkamp[7], Zoë Waller[5], Luca Magnani[4], John S. Schneekloth, Jr[64], Marco Di Antonio[1,2,75]*

Table of contents

Supplementary-1 Synthesis of Cl-G4 probes…....……..…………………………..............………......1

Supplementary-2 Synthesis of Cl-IM peptides ……………………...………………………….............12

Supplementary-3 Supplementary Tables…………………………………………………………......13

Supplementary-4 Supplementary Figures……………………………………….....…………….…........25

Supplementary-5 Supplementary plasmid sequence…………………………...……….……………........37

Supplementary-6 NMR Spectra.. 41

Supplementary-7 Peptides LC-MS characterizations....………………………...……….………….......50

Supplementary-8 Cl-pep-RVS$_n$ i-motif binding characterization *via* UV-melting...................................53

Supplementary-1 Synthesis of Cl-G4 probes

LCMS was performed on an Agilent 1260 Infinity Series HPLC, equipped with an Agilent Poroshell HPH C-18 3.0x50 mm 2.7 µm Column, connected to an Agilent LC/MSD XT Single Quadrupole Mass Spectrometer. The following solvent system was used with a flowrate of 0.8 mL/min: 0.1% HCOOH in mQ (A) and 0.1% HCOOH in 90:10 MeCN:mQ. Gradient: 95% A to 100% B in 7 min, followed by 1 min flushing with B (LCMS1) and 2 min equilibration with 95% A (**LCMS1**). HRMS was performed on an Agilent 6546 LC/Q-TOF High-Resolution MS spectrometer. The analyzed samples were solubilized on a LC-MS grade Solvent (MeOH) before each analysis at 0.05 mg/ml concentration.
NMR spectra were recorded on either a Bruker Avance III 400MHz or 500MHz spectrometer. All δ-values are expressed in ppm relative to CDCl3 (7.29 ppm for proton and 76.9 ppm for carbon) or DMSO-d6 (2.50 ppm for proton and 39.5 ppm for carbon). The following abbreviations explain the multiplicities: s=singlet, d=doublet, t=triplet, q=quartet, m=multiplet, and br=broad.

Synthesis of ligands **PyPDs-3TFA**[1], **PhenDC3-NH_2**[2], **PDC-NH_2** [3] and **Cl-PEG2-COOH**[4]was performed according to previous reports.

General synthetic route for Cl-PyPDSn, Cl-PhenDC3n and Cl-PDC$_2$

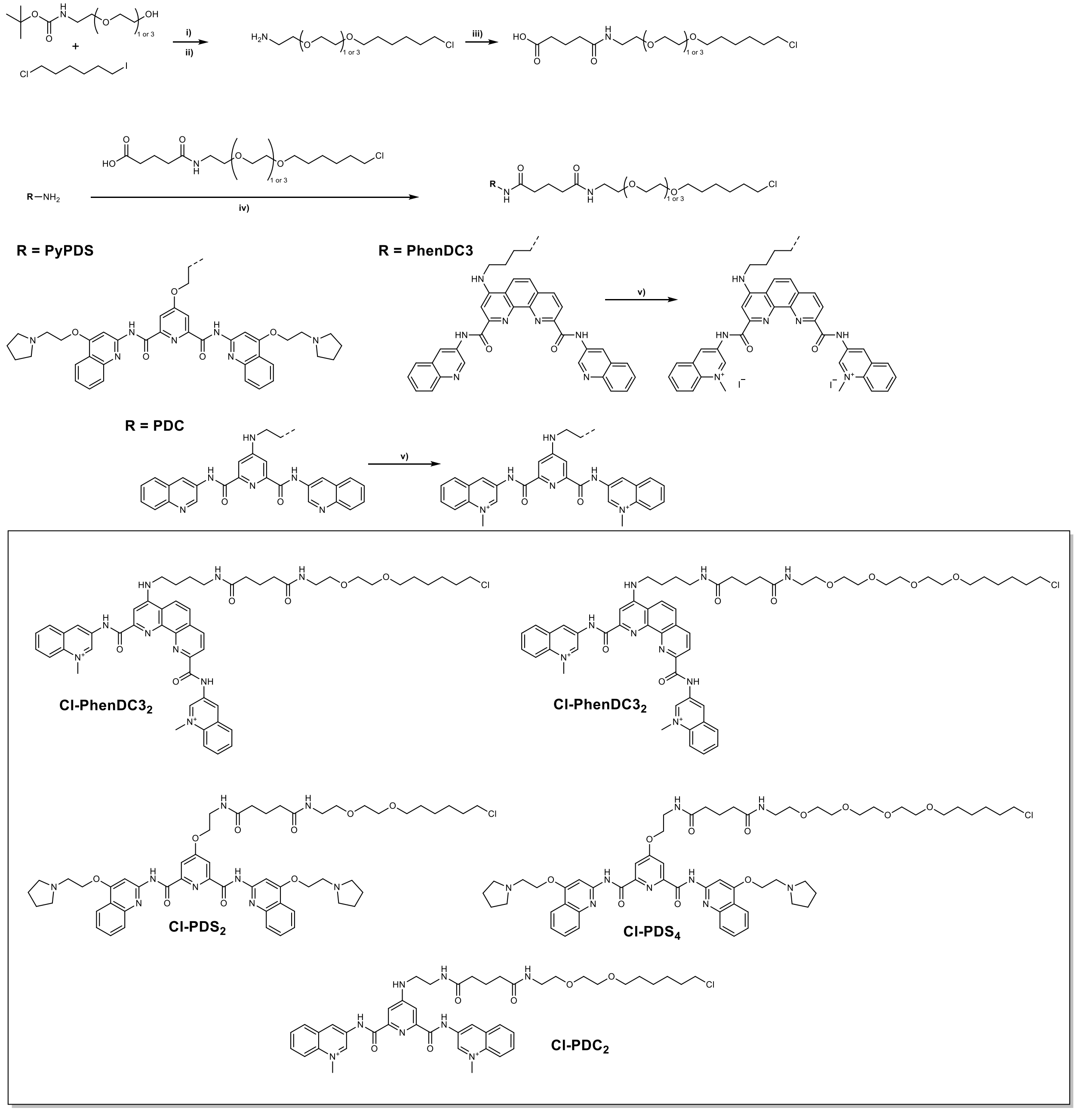

Supplementary Figure 1 Synthetic route for compounds Cl-PyPDS$_n$, Cl-PhenDC3$_n$ and Cl-PDC$_2$. **i)** NaH, DMF:THF (1:2), 0°C to RT, 16h; **ii)** TFA, DCM**,** 2h, 0°C; **iii)** DCM, Glutaric anhydride, DMAP, DIPEA, 2h, rt; **iv)** EDC-Cl, HOBt, DIPEA DMF, 16h, rt; **v)** CH3I, DMF, on, rt.

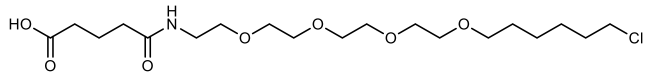

24-chloro-5-oxo-9,12,15,18-tetraoxa-6-azatetracosanoic acid **(Cl-PEG4-COOH)**

Compound **Cl-PEG4-COOH** was synthesized by adapting previous synthetic reports for the synthesis of **Cl-PEG2-COOH**, using 18-chloro-3,6,9,12-tetraoxaoctadecan-1-amine as the starting material. To a solution of dry DCM containing glutaric anhydride (20 mg, 0.175 mmol), DMAP (19.2 mg. 0.16 mmol), DIPEA (167 µL, 0.963 mmol) and finally 18-chloro-3,6,9,12-tetraoxaoctadecan-1-amine (60 mg, 0.193 mmol), were subsequently added and allowed to react under inert atmosphere for 2h. The reaction was monitored through TLC, using DCM:MeOH 9:1 as the mobile phase. Upon reaction completion, the mixture was diluted with EtOAc to 50 mL, washed three times with 25 mL HCl 1M, and once with 25 mL brine. The organic layer was dried over anhydrous Na_2SO_4 and concentrated in vacuo to obtain compound **Cl-PEG4-COOH** with 75.4% yield (56 mg, 0.132 mmol).

^{1}H NMR (400 MHz, $CDCl_3$) δ 3.72 – 3.42 (m, 20H), 2.40 (q, J = 6.8 Hz, 4H), 1.97 (p, J = 7.0 Hz, 2H), 1.76 (p, J = 6.8 Hz, 2H), 1.60 (p, J = 6.9 Hz, 2H), 1.44 (dt, J = 14.3, 6.8 Hz, 2H), 1.35 (dt, J = 14.2, 6.3 Hz, 2H). **^{13}C NMR** (101 MHz, $CDCl_3$) δ 173.48, 172.66, 71.31, 70.48, 70.40, 70.32, 70.01, 69.92, 51.67, 50.80, 45.05, 39.23, 32.98, 32.80, 29.23, 26.65, 25.36, 20.90, 19.90. **HRMS** (MeOH) calcd. For $C_{19}H_{35}ClN_1O_7^-$ $[M-H]^-$ 424.2108; found: 424.2121.

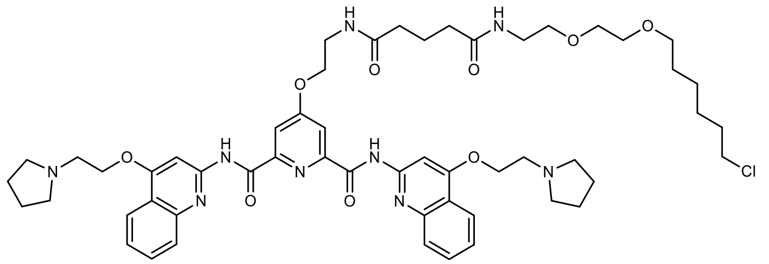

4-((21-chloro-4,8-dioxo-12,15-dioxa-3,9-diazahenicosyl)oxy)-N2,N6-bis(4-(2-(pyrrolidin-1-yl)ethoxy)quinolin-2-yl)pyridine-2,6-dicarboxamide (**Cl-PyPDS$_2$**)

In a 5ml round-bottomed flask, compound **Cl-PEG2-COOH** (19.6 mg, 0.058 mmol) was solubilized in 1 mL of dry DMF and stirred under inert conditions. To the solution, EDC-Cl (11.2 mg, 0.058 mmol), HOBt (0.9 mg, 0.006 mmol), and DIPEA (10.1 µL, 0.058 mmol) were added in this order, and the mixture allowed to react for 2 minutes at room temperature. Subsequently, **PyPDS-3TFA** (30 mg, 0.029 mmol) was added to the mixture, the pale-yellow suspension obtained was stirred overnight under an inert atmosphere, and the completion was checked via TLC (10% MeOH, 1% TEA in DCM). To the clear solution obtained, 25 mL of water was added, and the resulting white precipitate was centrifuged and washed 3 times with water to obtain the titled compound **Cl-PyPDS$_2$** with 71.5% yield (21.2 mg, 0.0207 mmol).

^{1}H-NMR (400 MHz, DMSO) δ 12.05 (s, 2H), 8.16 – 8.09 (m, 5H), 7.98 – 7.89 (m, 4H), 7.80 (dt, *J* = 24.8, 6.9 Hz, 3H), 7.54 (t, *J* = 7.5 Hz, 2H), 4.42 (t, *J* = 5.5 Hz, 4H), 4.32 (t, *J* = 5.7 Hz, 2H), 3.59 (t, *J* = 6.6 Hz, 2H), 3.52 (q, *J* = 5.6 Hz, 2H), 3.46 – 3.30 (m, 6H*), 3.17 (q, *J* = 6.0 Hz, 2H), 3.02 (t, *J* = 5.5 Hz, 4H), 2.68 – 2.60 (m, 8H), 2.09 (dt, *J* = 15.0, 7.5 Hz, 4H), 1.77 – 1.62 (m, 12H), 1.48 – 1.23 (m, 8H). * Peak partially overlapping with H_2O. **^{13}C-NMR** (101 MHz, DMSO) δ 172.67, 172.24, 167.57, 163.69, 162.57, 152.94, 151.55, 147.54, 131.05, 127.39, 125.16, 122.20, 119.70, 112.56, 95.46, 70.62, 70.00, 69.86, 69.59, 68.54, 68.00, 54.69, 54.29, 45.80, 38.90, 38.16, 35.10, 32.46, 29.50, 26.57, 25.37, 23.73, 21.90. **LCMS** (ESI, **LCMS1** conditions, RT=3.95 min): 1024.5. **HRMS** (ESI, MeOH) calcd. For $C_{54}H_{71}ClN_9O_9^+$ $[M+H]^+$ 1024.5058; found: 1024.5050.

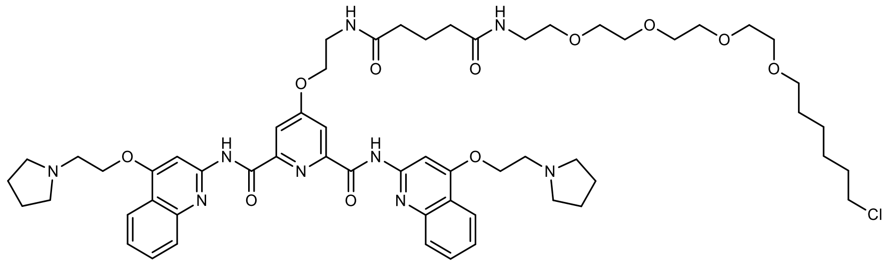

4-((27-chloro-4,8-dioxo-12,15,18,21-tetraoxa-3,9-diazaheptacosyl)oxy)-N2,N6-bis(4-(2-(pyrrolidin-1-yl)ethoxy)quinolin-2-yl)pyridine-2,6-dicarboxamide (**Cl-PyPDS$_4$**)

In a 5ml round-bottomed flask, compound **Cl-PEG4-COOH** (24.8 mg, 0.058 mmol) was solubilized in 1 mL of dry DMF and stirred under inert conditions. To the solution, EDC-Cl (11.2 mg, 0.058 mmol), HOBt (0.9 mg, 0.006 mmol) and DIPEA (10.1 µL, 0.058 mmol) were added in this order, and the mixture allowed to react for 2 minutes at room temperature. Subsequently, **PyPDS-3TFA** (30 mg, 0.029 mmol), was added to the mixture, the pale-yellow suspension obtained was stirred overnight under inert atmosphere, and the completion was checked via TLC (10% MeOH, 1% TEA in DCM). To the clear solution obtained, 25 mL of water were added, and the resulting white precipitate was centrifuged and washed 3 times with water to obtain the titled compound **Cl-PyPDS$_4$** with 72.2 % yield (23.1 mg, 0.0210 mmol).

^{1}H-NMR (400 MHz, DMSO) δ 12.05 (s, 2H), 8.13 (d, *J* = 8.2 Hz, 5H), 7.97 – 7.90 (m, 4H), 7.85 (t, *J* = 5.6 Hz, 1H), 7.78 (ddd, *J* = 8.5, 6.9, 1.5 Hz, 2H), 7.55 (t, *J* = 7.6 Hz, 2H), 4.42 (t, *J* = 5.5 Hz, 4H), 4.33 (t, *J* = 5.5 Hz, 2H), 3.60 (t, *J* = 6.6 Hz, 4H), 3.55 – 3.30 (m, 14H*), , 3.18 (dd, *J* = 7.7, 3.8 Hz, 2H), 3.03 (t, *J* = 5.5 Hz, 4H), 2.70 – 2.59 (m, 8H), 2.10 (dt, *J* = 14.6, 7.4 Hz, 4H), 1.80 – 1.64 (m, 12H), 1.50 – 1.21 (m, 8H). * Peak partially overlapping with H_2O. **^{13}C-NMR** (101 MHz, DMSO) δ 172.23, 172.00, 167.11, 163.21, 162.11, 152.47, 151.07, 147.07, 130.59, 126.94, 124.70, 121.76, 119.24, 112.09, 94.99, 70.17, 69.79, 69.73, 69.58, 69.47, 69.14, 68.07, 67.48, 54.24, 53.84, 51.25, 45.36, 38.47, 37.75, 34.65, 34.23, 33.43, 32.59, 32.03, 29.06, 26.12, 24.93, 23.27, 21.45, 20.58. **LCMS** (ESI, **LCMS1** conditions, RT=3.98 min): 1112.5. **HRMS** (ESI, MeOH) calcd. For $C_{58}H_{79}ClN_9O_{11}^+$ $[M+H]^+$ 1112.5582; found: 1112.5576.

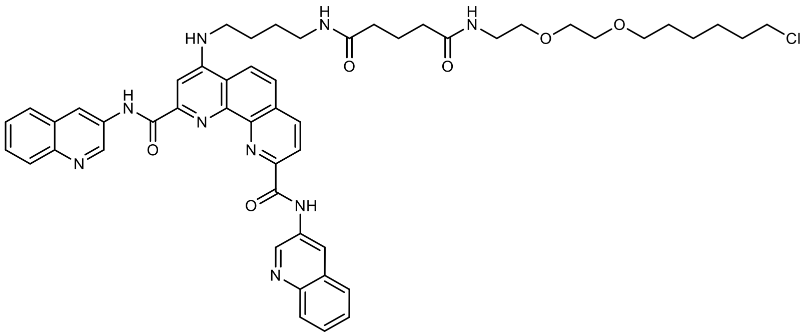

4-((23-chloro-6,10-dioxo-14,17-dioxa-5,11-diazatricosyl)amino)-N2,N9-di(quinolin-3-yl)-1,10-phenanthroline-2,9-dicarboxamide (**S1_Cl-PhenDC3$_2$**)

In a 5ml round-bottomed flask, compound **Cl-PEG2-COOH** (13 mg, 0.039 mmol) was solubilized in 1 mL of dry DMF and stirred under inert conditions. To the solution, EDC-Cl (18.5 mg, 0.077 mmol), HOBt (1.3 mg, 0.01 mmol), and DIPEA (27 µL, 0.150 mmol) were added in this order, and the mixture was allowed to react for 2 minutes at room temperature. Subsequently, **PhenDC3-NH$_2$** (25 mg, 0.047 mmol) was added to the mixture, the pale-yellow suspension obtained was stirred overnight under an inert atmosphere, and the completion was checked via TLC (10% MeOH, 1% TEA in DCM). To the clear solution obtained, 25 mL of water was added, and the resulting white precipitate was centrifuged and washed 3 times with water to obtain the titled compound **S1_Cl-PhenDC3$_2$** with 80.1 % yield (28.9 mg, 0.033 mmol).

^{1}H-NMR (400 MHz, DMSO) δ 11.78 (s, 2H), 9.68 (s, 2H), 9.12 (d, *J* = 11.7 Hz, 2H), 8.78 (d, *J* = 8.6 Hz, 1H), 8.62 – 8.46 (m, 2H), 8.08 (d, *J* = 9.8 Hz, 5H), 7.94 – 7.52 (m, 8H), 3.62 – 3.09 (m, 18H), 2.23 – 1.95 (m, 4H), 1.69 (m, 8H), 1.54 – 1.06 (m, 4H). **^{13}C-NMR** (126 MHz, DMSO) δ 173.04, 171.78, 171.69, 171.38, 163.68, 152.31, 147.93, 145.67, 138.80, 135.46, 134.69, 134.44, 133.84, 132.75, 130.91, 130.25, 129.91, 129.11, 125.27, 122.86, 121.60, 119.20, 99.60, 70.13, 69.75, 69.68, 69.53, 69.44, 69.10, 52.45, 52.29, 51.21, 46.31, 46.09, 45.34, 38.69, 38.40, 38.08, 36.10, 35.46, 34.86, 34.71, 34.37, 34.33, 33.03, 32.81, 32.66, 31.98, 29.01, 27.00, 26.98, 26.17, 26.07, 25.16, 24.88, 21.59, 20.66. **LCMS** (ESI, **LCMS1** conditions, RT=5.60 min): 926.4. **HRMS** (ESI, MeOH) calcd. For $C_{51}H_{57}ClN_9O_6^+$ $[M+H]^+$ 926.4115; found: 926.4111.

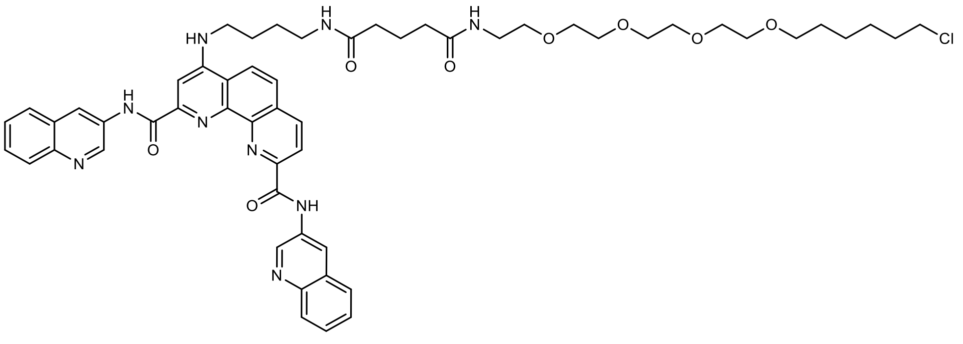

4-((29-chloro-6,10-dioxo-14,17,20,23-tetraoxa-5,11-diazanonacosyl)amino)-N2,N9-di(quinolin-3-yl)-1,10-phenanthroline-2,9-dicarboxamide (**S2_Cl-PhenDC3$_4$**)

In a 5ml round-bottomed flask, compound **Cl-PEG4-COOH** (16.6 mg, 0.039 mmol) was solubilized in 1 mL of dry DMF and stirred under inert conditions. To the solution, EDC-Cl (18.5 mg, 0.077 mmol), HOBt (1.3 mg, 0.01 mmol), and DIPEA (27 µL, 0.150 mmol) were added in this order, and the mixture was allowed to react for 2 minutes at room temperature. Subsequently, **PhenDC3-NH$_2$** (25 mg, 0.047 mmol) was added to the mixture, the pale-yellow suspension obtained was stirred overnight under an inert atmosphere, and the completion was checked via TLC (10% MeOH, 1% TEA in DCM). To the clear solution obtained, 25 mL of water was added, and the resulting white precipitate was centrifuged and washed 3 times with water to obtain the titled compound **S2_Cl-PhenDC3$_4$** with 85.3 % yield (34.4 mg, 0.034 mmol).

^{1}H NMR (400 MHz, DMSO) δ 11.78 (s, 2H), 9.68 (s, 2H), 9.12 (d, *J* = 11.7 Hz, 2H), 8.78 (d, *J* = 8.6 Hz, 1H), 8.62 – 8.46 (m, 2H), 8.08 (d, *J* = 9.8 Hz, 5H), 7.94 – 7.58 (m, 8H), 3.75 – 3.13 (m, 22H), 2.36 – 2.23 (m, 2H), 2.19 – 1.95 (m, 4H), 1.69 (d, *J* = 60.8 Hz, 8H), 1.54 – 1.06 (m, 4H).
^{13}C -NMR (126 MHz, DMSO) δ 171.82, 171.68, 164.15, 163.57, 152.18, 149.60, 148.95, 145.48, 145.40, 144.65, 144.58, 144.51, 144.19, 138.25, 132.56, 132.51, 130.38, 128.68, 128.26, 128.16, 128.04, 127.98, 127.84, 127.14, 127.10, 124.74, 123.56, 123.28, 122.56, 121.10, 119.11, 99.17, 70.15, 69.77, 69.70, 69.55, 69.45, 69.13, 45.34, 42.61, 38.44, 38.15, 34.85, 34.73, 32.00, 29.03, 27.02, 26.09, 25.29, 24.90, 22.09, 21.60. **LCMS** (ESI, **LCMS1** conditions, RT=5.36 min): 1014.4. **HRMS** (ESI, MeOH) calcd. For $C_{51}H_{57}ClN_9O_6^+$ [M+H]$^+$ 1014.4639; found: 1014.4630.

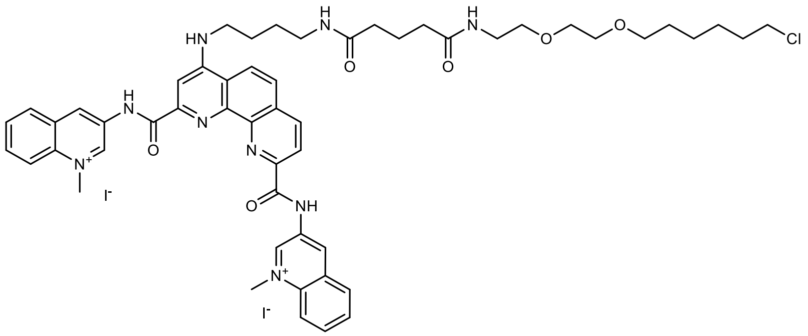

3,3'-((4-((23-chloro-6,10-dioxo-14,17-dioxa-5,11-diazatricosyl)amino)-1,10-phenanthroline-2,9-dicarbonyl)bis(azanediyl))bis(1-methylquinolin-1-ium) diiodide (**Cl-PhenDC3₂**)

In a 5 mL pressure vessel, compound **S1_Cl-PhenDC3₂** (25 mg, 0.027 mmol) was suspended in 1.8 mL of dry DMF. Subsequently, methyl iodide (100 µL, 1.6 mmol) was added to the mixture and stirred overnight at 40°C. The reaction was poured into diethyl ether (25 mL) upon completion. The yellow precipitate obtained was filtered, washed 3 times with diethyl ether, and dried to obtain compound **Cl-PhenDC3₂** as iodide salt in quantitative yield (32.5 mg, 0.027 mmol).

^{1}H NMR (500 MHz, DMSO) δ 12.12 (s, 2H), 10.29 (d, *J* = 2.3 Hz, 2H), 9.87 (s, 2H), 8.87 (d, *J* = 8.1 Hz, 1H), 8.68 (dd, *J* = 8.3, 4.6 Hz, 1H), 8.61 (d, *J* = 9.3 Hz, 1H), 8.58 – 8.45 (m, 5H), 8.27 – 8.17 (m, 4H), 8.06 (td, *J* = 7.7, 3.0 Hz, 3H), 7.84 (dt, *J* = 11.9, 5.7 Hz, 2H), 7.69 (d, *J* = 7.4 Hz, 1H), 4.72 (d, *J* = 5.0 Hz, 6H), 3.59 (t, *J* = 6.6 Hz, 2H), 3.50-3.30 (m, 6H), 3.18 – 3.09 (m, 6H), 2.54 (t, *J* = 5.6 Hz, 4H), 2.06 (t, *J* = 7.5 Hz, 4H), 1.80 – 1.58 (m, 8H), 1.44 (q, *J* = 7.0 Hz, 2H), 1.38 – 1.23 (m, 4H). **^{13}C NMR** (126 MHz, DMSO) δ 171.79, 171.69, 163.70, 147.94, 145.70, 138.81, 135.46, 135.41, 134.70, 134.45, 133.85, 132.77, 130.91, 130.25, 130.23, 129.93, 129.90, 129.13, 129.11, 125.28, 122.88, 121.58, 119.20, 99.58, 70.14, 69.52, 69.38, 69.09, 46.08, 46.06, 45.34, 38.41, 38.07, 34.86, 34.73, 34.37, 31.97, 29.02, 27.00, 26.08, 25.16, 24.88, 21.60. **LCMS** (ESI, **LCMS1** conditions, RT=3.57 min): 954.4. **HRMS** (ESI, MeOH) calcd. For $C_{53}H_{61}ClN_9O_6^+$ $[M+H]^+$ 954.4428; found: 954.4419.

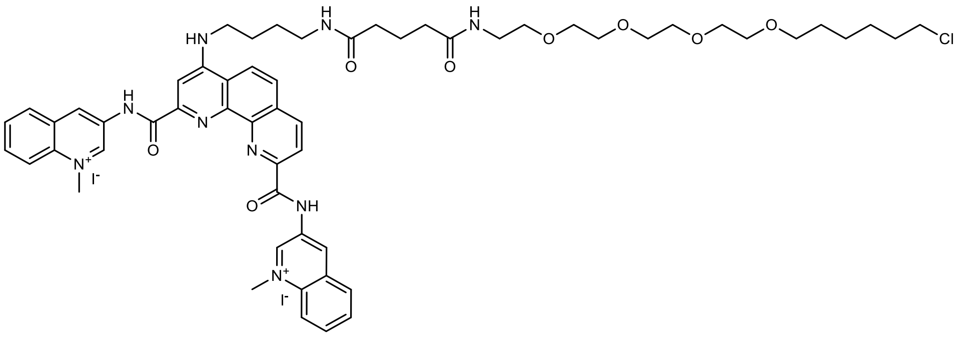

3,3'-((4-((29-chloro-6,10-dioxo-14,17,20,23-tetraoxa-5,11-diazanonacosyl)amino)-1,10-phenanthroline-2,9-dicarbonyl)bis(azanediyl))bis(1-methylquinolin-1-ium) diiodide (**Cl-PhenDC3₄**)

In a 5 mL pressure vessel, compound **S2_Cl-PhenDC3₄** (25 mg, 0.0247 mmol) was suspended in 1.8 mL of dry DMF. Subsequently, methyl iodide (92 µL, 1.48 mmol) was added to the mixture and stirred overnight at 40°C. The reaction was poured into diethyl ether (25 mL) upon completion. The yellow precipitate obtained was filtered, washed 3 times with diethyl ether, and dried to obtain compound **Cl-PhenDC3₄** as iodide salt in quantitative yield (32 mg, 0.0247 mmol).

^{1}H NMR (500 MHz, DMSO) δ 12.11 (s, 2H), 10.29 (s, 2H), 9.93 – 9.78 (m, 2H), 8.87 (d, *J* = 7.9 Hz, 1H), 8.70 – 8.60 (m, 2H), 8.56 – 8.47 (m, 4H), 8.23 (q, *J* = 9.2 Hz, 4H), 8.10 – 8.04 (m, 2H), 7.85 (d, *J* = 25.6 Hz, 2H), 7.74 – 7.63 (m, 2H), 4.72 (d, *J* = 5.3 Hz, 6H), 3.45 (m, 18H), 3.18 – 3.09 (m, 4H), 2.54 (t, *J* = 5.6 Hz, 3H), 2.28 (q, *J* = 6.5 Hz, 1H), 2.08 (m, 3H), 1.81 – 1.56 (m, 8H), 1.57 – 1.18 (m, 4H). **^{13}C-NMR** (126 MHz, DMSO) δ 173.04, 171.78, 171.69, 171.38, 163.68, 152.31, 147.93, 145.67, 138.80, 135.46, 134.69, 134.44, 133.84, 132.75, 130.91, 130.25, 129.91, 129.11, 125.27, 122.86, 121.60, 119.20, 99.60, 70.13, 69.75, 69.68, 69.53, 69.44, 69.10, 51.21, 46.09, 45.34, 38.40, 38.08, 34.86, 34.71, 34.37, 32.66, 31.98, 29.01, 27.00, 26.98, 26.17, 26.07, 25.16, 24.88, 21.59, 20.66. **LCMS** (ESI, **LCMS1** conditions, RT=3.64 min): 1042.4. **HRMS** (ESI, MeOH) calcd. For $C_{57}H_{69}ClN_9O_8^+$ $[M+H]^+$ 1042.4952; found: 1042.4950.

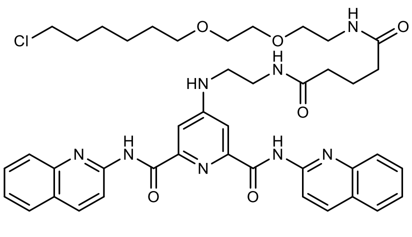

4-((21-chloro-4,8-dioxo-12,15-dioxa-3,9-diazahenicosyl)amino)-N2,N6-di(quinolin-2-yl)pyridine-2,6-dicarboxamide **(S3_Cl-PDC₂)**

In a 5ml round-bottomed flask, compound **Cl-PEG2-COOH** (40 mg, 0.120 mmol) was solubilized in 1 mL of dry DMF and stirred under inert conditions. To the solution, EDC-Cl (23.2 mg, 0.120 mmol), HOBt (1.8 mg, 0.012 mmol), and DIPEA (20.8 µL, 0.116 mmol) were added in this order, and the mixture was allowed to react for 2 minutes at room temperature. Subsequently, **PDC-NH₂** (28.8 mg, 0.060 mmol) was added to the mixture, the pale-yellow suspension obtained was stirred overnight under an inert atmosphere, and the completion was checked via TLC (10% MeOH, 1% TEA in DCM). To the clear solution obtained, 30 mL

of water were added, and the resulting white precipitate was centrifuged and washed 3 times with water to obtain the titled compound **S3_Cl-PDC₂** with 88.2% yield (27.6 mg, 0.046 mmol).

¹H NMR (400 MHz, DMSO) δ 11.32 (br, 2H), 9.36 (d, J = 2.4 Hz, 2H), 8.95 (dd, J = 12.2, 2.2 Hz, 2H), 8.05 – 7.99 (m, 5H), 7.84 (t, J = 5.5 Hz, 1H), 7.73 – 7.56 (m, 7H), 3.65 – 3.53 (m, 2H), 3.49 – 3.41 (m, 4H), 3.36 – 3.30 (m, 9H), 3.18 – 3.09 (m, 2H), 2.12 – 2.04 (m, 4H), 1.68 (dtd, J = 25.8, 13.0, 7.0 Hz, 4H), 1.45 (dt, J = 13.9, 6.7 Hz, 2H), 1.41 – 1.18 (m, 4H). **¹³C NMR** (101 MHz, DMSO) δ 172.63, 172.28, 163.60, 156.82, 146.55, 145.11, 132.47, 129.15, 128.76, 128.35, 128.32, 128.22, 128.19, 127.62, 124.57, 70.64, 70.01, 69.87, 69.60, 45.82, 45.19, 41.97, 38.92, 35.26, 35.17, 32.47, 29.51, 26.58, 25.38, 21.87. **HRMS** (ESI, MeOH) calcd. For $C_{42}H_{50}ClN_8O_6^+$ $[M+H]^+$ 797.3536; found: 797.3525.

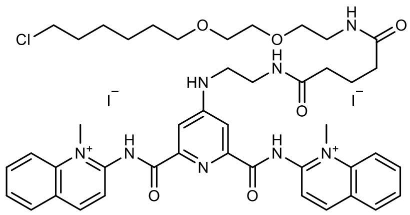

2,2'-((4-((21-chloro-4,8-dioxo-12,15-dioxa-3,9-diazahenicosyl)amino)pyridine-2,6-dicarbonyl)bis(azanediyl))bis(1-methylquinolin-1-ium) iodide (Cl-PDC₂)

In a 5 mL pressure vessel, compound **S3_Cl-PDC₂** (15 mg, 0.019 mmol) was suspended in 1.0 mL of dry DMF. Subsequently, methyl iodide (80 µL, 1.28 mmol) was added to the mixture and stirred overnight at room temperature. The reaction was poured into diethyl ether (25 mL) upon completion. The yellow precipitate obtained was filtered, washed 3 times with diethyl ether, and dried to obtain compound **Cl-PDC₂** as iodide salt in quantitative yield (20 mg, 0.019 mmol).

¹H NMR (400 MHz, DMSO) δ 11.77 – 11.66 (m, 2H), 10.13 (s, 2H), 9.66 (d, *J* = 10.2 Hz, 2H), 8.55 (d, *J* = 5.9 Hz, 4H), 8.15 (dt, *J* = 61.2, 7.1 Hz, 4H), 7.87 – 7.71 (m, 2H), 7.58 (dd, *J* = 32.3, 6.3 Hz, 4H), 4.78 (s, 6H), 3.65 – 3.08 (m, 16H), 2.18 – 1.96 (m, 4H), 1.71 (dq, *J* = 14.5, 7.2 Hz, 4H), 1.58 – 1.40 (m, 2H), 1.42 – 1.14 (m, 4H). **¹³C NMR** (101 MHz, DMSO) δ 172.70, 172.30, 163.64, 157.17, 145.20, 136.16, 134.63, 134.49, 134.41, 132.89, 132.77, 130.84, 130.35, 129.71, 119.70, 70.65, 70.03, 69.88, 69.59, 46.65, 45.85, 41.94, 38.92, 38.09, 35.30, 35.18, 32.47, 29.51, 26.58, 25.38, 21.88. **HRMS** (ESI, MeOH) calcd. For $C_{44}H_{56}ClN_8O_6^+$ $[M+H]^+$ 827.3994; found: 827.3985.

Supplementary-2 Synthesis of Cl-pep-RVS$_n$

The synthesis of the iM-binding peptides was performed according to standard Fmoc-SPPS procedures on an Intavis MultiPep peptide synthesiser, using HATU/DIPEA as coupling reagents, each coupling cycle consisting of 5eq of Fmoc-AA, 4.8eq of HATU and 10eq of DIPEA. Coupling step was repeated twice for 30 minutes to ensure the completion of the reaction. Loading of the first amino acid was performed manually on a Wang resin, using 5 eq of amino acid (Fmoc-Gly-OH), 5eq of HOBt, 0.2 eq DMAP and 5 of DIC. The loading of the resin was calculated before starting the synthesis on the synthesizer. For further functionalization of the peptide with the chloroalkane tag (**Cl-pep-RVS$_2$** and **Cl-pep-RVS$_4$,** see **Scheme 2** below), the peptide was firstly functionalized with succinic anhydride (10 eq succinic anhydride + 10 eq of DIPEA in DMF, 45 min). The obtained carboxylic acid was therefore pre-activated with a mixture of HATU and DIPEA (10eq HATU and 20 DIPEA with respect to the resin) for 2 minutes. HATU was chosen to reduce the risk of epimerization. Subsequently, a solution contain 2-(2-((6-Chlorohexyl)oxy)ethoxy)ethanamine hydrochloride(2-(2-(6-chlorohexyloxy)ethoxy)ethanamine HCl) (10eq), was added to the pre-activated resin, and coupled for 2h, shaken vigorously. The obtained chloro-alkane functionalised peptides, as well as the control peptide **pep-RVS**, were cleaved using a mixture of TFA containing 2% H_2O and 2% Triisopropylsilane (TIS) as scavengers. The excess TFA was evaporated under a flow of nitrogen, the peptides were precipitated in Et_2O, centrifuged, and the supernatant discarded. The residue was re-suspended in Et_2O and the washing procedure repeated twice. The obtained crude peptides were purified on an Agilent Series 1200 HPLC-UV instrument, equipped with a Phenomenex Luna C18(2) (5 µm, 100 Å, 250x4.6 mm), with the following gradient: 100% mQ + 0.1% TFA for 5 min, then a gradient from 0 to 30% MeCN over 18 min at a flow rate of 4.0 ml/min. Purity and identity of the peptides was assessed by HPLC-MS, on an Agilent 1100 Series instrument equipped with a Phenomenex Kinetex C18 100 Å column (150 x 4.6 mm, 5 µm at 35 °C) connected to an ESMSD type VL mass detector (quadrupole ion trap mass spectrometer) with a flow rate of 1.5 ml/min was used with the following solvent systems: (A) 0.1% Formic Acid (HCOOH) in mQ and (B) MeCN. Gradient: 100% A for 2 min, then a gradient from 0 to 100% B over 6 min was used, followed by 2 min of flushing with 100% B.

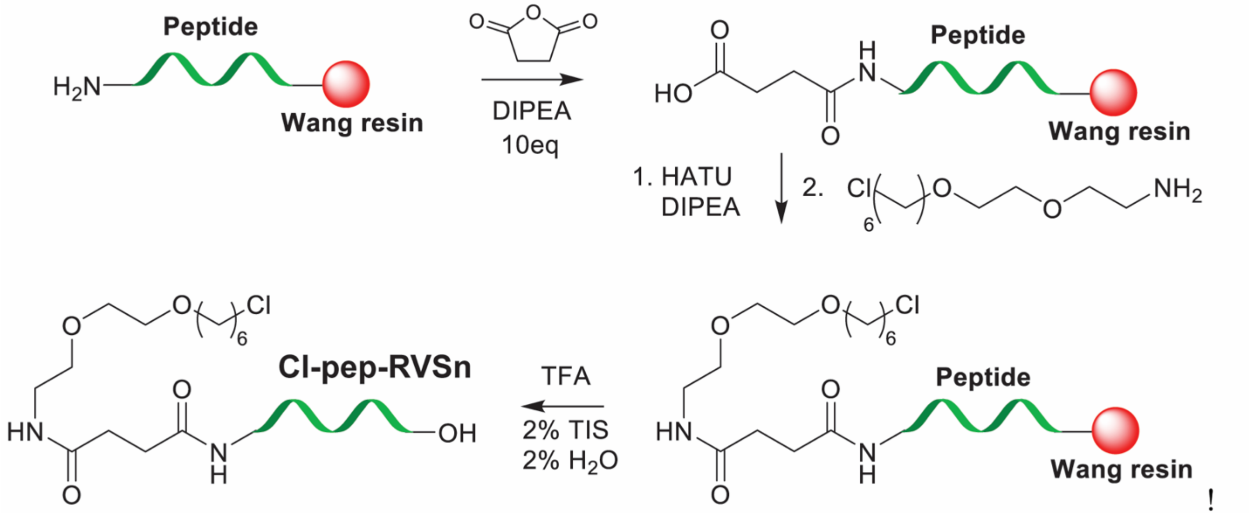

Supplementary Figure 2. Synthetic route for the synthesis of **Cl-pep-RVS$_2$** and **Cl-pep-RVS$_4$** directly on solid support.

Supplementary-3 Supplementary tables

Supplementary Table 1. Oligos for FRET assay, CD melting and UV-melting

All the oligonucleotides used for the FRET melting assay and dCas9_FRET assay oligonucleotides were purchased from Integrated DNA Technologies (IDT) and HPLC purified. Stock solutions of 100 µM were prepared in UltraPure™ DNase/RNase-Free Distilled Water.

**Name**	**Sequence (5'-3')**
**BCL-2**	FAM-GGGCGCGGGAGGAAGGGGGCGGG-TAMRA
**hTELO**	FAM-GGGTTAGGGTTAGGGTTAGGG-TAMRA
**c-KIT2**	FAM-CGGGCGGGCGCGAGGGAGGGG-TAMRA
**c-MYC-Pu22**	TGAGGGTGGGTAGGGTGGGTAA
**c-MYC-C52**	CTTCTCCCCACCTTCCCCACCCTCCCCACCCTCCCCATAAGCGCCCCT CCCG
**c-MYC-G52**	CGGGAGGGGCGCTTATGGGGAGGGTGGGGAGGGTGGGGAAGGTGGG GAGAAG
**dsDNA_26mer**	FAM-CAATCGGATCGAATTCGATCCGATTG-TAMRA
**dCas9_FRET.Fw**	TCCTCCTCCCGGCGGGCACAGCCCCCCGGCATTAACACGTCAGACGC CGCCGGGAAGAAGCGAGACCCGGGCGGGCGCGAGGGAGGGGA_Cy5
**dCas9_FRET.Rev**	Cy3_GTCTCGCTTCTTCCCGGCGGCGTCTGACGTGTTAATGCCGGGGG GCTGTGCCCGCCGGGAGGAGGA

Supplementary Table 2. sgRNA sequences for dCas9_FRET assay

All the oligonucleotides used for the dCas9_FRET assay were purchased from IDT and purified by standard desalting. Stock solutions of 100 µM were prepared in UltraPure™ DNase/RNase-Free Distilled Water.

**Name**	**Protospacer (5'-3')**	**Sequence (5'-3') for IVT**
**NT-sgRNA $_{FRET-18}$**	ATTAACACGTCAGACGCCGC	TAATACGACTCACTATAGATTAA CACGTCAGACGCCGC GTTTCAGAGCTATCTGGAAAC
**NT-sgRNA $_{FRET-42}$**	TCCCGGCGGGCACAGCCCCC	TAATACGACTCACTATAGTCCCG GCGGGCACAGCCCCC GTTTCAGAGCTATCTGGAAAC
**T-sgRNA $_{FRET-41}$**	GGGGGGCTGTGCCCGCCGGG	TAATACGACTCACTATAGGGGG GCTGTGCCCGCCGGGGTTTCAG AGCTATCTGGAAAC

**T-sgRNA** $_{\text{FRET-21}}$	GCGTCTGACGTGTTAATGCC	TAATACGACTCACTATAGCGTCTGACGTGTTAATGCCGTTTCAGAGCTATCTGGAAAC
**sgRNA** $_{\text{FRET NTC}}$	CGCCCAAGTGCAGCGAGCGC	TAATACGACTCACTATACGCCCAAGTGCAGCGAGCGC GTTTCAGAGCTATGCTGGAAAC

Supplementary Table 3. Cloning and genotyping primer sequence

**Name**	**Sequence (5'-3')**
**Lenti_Halotag.Fw**	gaggaaggtggcggccgctggatccGCAGAAATCGGTACTGGCTTTC
**Lenti_Halotag.Rev**	ctctgccctctccactgcctgtacAACCGGAAATCTCCAGAGTAGA
**Lenti_T2A_mCherry. Fw**	TACTCTGGAGATTTCCGGTTgtacagagggcagaggaagtctg
**Lenti_T2A_mCherry. Rev**	taccgataagcttgatatcGAATTCTTACTTGTACAGCTCG
**SC_dCas9-genotyping.Fw**	gtacggccaccatgaaaag
**SC_dCas9-genotyping.Rev**	atgttagcagacttcctct

Supplementary Table 4. sgRNA sequences for cloning in pLG1 backbone

Name	Protospacer sequence	Fw primer sequence (5'-3')	Rev primer sequence (5'-3')
**MYC-19**	AGCTAGAGTGCTCGGCTGCC	TTGGAGCTAGAGTGCTCGGCTGCCGTTTAAGAGC	TTAGCTCTTAAACGGCAGCCGAGCACTCTAGCTCCAACAag
**MYC-67**	GCGCGCGTAGTTAATTCATG	TTGGCGCGCGTAGTTAATTCATGGTTTAAGAGC	TTAGCTCTTAAACCATGAATTAACTACGCGCGCCAACAag
**MYC-89**	AGGGAGCAAAAGAAAATGGT	TTGGAGGGAGCAAAAGAAAATGGTGTTTAAGAGC	TTAGCTCTTAAACACCATTTTCTTTTGCTCCCTCCAACAag
**MYC-93**	TGAGAGGGAGCAAAAGAAAA	TTGGTGAGAGGGAGCAAAAGAAAAGTTTAAGAGC	TTAGCTCTTAAACTTTTCTTTTGCTCCCTCTCACCAACAag
**MYC+22**	CCCCCACGCCCTCTGCTTT	TTGGCCCCCACGCCCTCTGCTTTGTTTAAGAGC	TTAGCTCTTAAACAAAGCAGAGGGCGTGGGGGCCAACAag
**MYC+58**	TGGGCGGAGATTAGCGAGAG	TTGGTGGGCGGAGATTAGCGAGAGGTTTAAGAGC	TTAGCTCTTAAACCTCTCGCTAATCTCCGCCCACCAACAag
**MYC+75**	CATTATAAAGGGCCGGTGGG	TTGGCATTATAAAGGGCCGGTGGGGTTTAAGAGC	TTAGCTCTTAAACCCCACCGGCCCTTTATAATGCCAACAag
**MYC+77**	TCGCATTATAAAGGGCCGGT	TTGGTCGCATTATAAAGGGCCGGTGTTTAAGAGC	TTAGCTCTTAAACACCGGCCCTTTATAATGCGACCAACAag
**MYC+119**	CCGCGAGCAGCACAGCTCGG	TTGGCCGCGAGCAGCACAGCTCGGGTTTAAGAGC	TTAGCTCTTAAACCCGAGCTGTGCTGCTCGCGGCCAACAag
**PVT1-20**	GGCTCAGTGCCCTGCGCTGC	TTGGGCTCAGTGCCCTGCGCTGCGTTTAAGAGC	TTAGCTCTTAAACGCAGCGCAGGGCACTGAGCCCAACAag
**PVT1+33**	GGGGAGGGGGGAGGCGCGCG	TTGGGGGAGGGGGGAGGCGCGCGGTTTAAGAGC	TTAGCTCTTAAACCGCGCGCCTCCCCCCTCCCCCAACAag
**HMNG1-22**	GCCCCCTTCGCCTGGGTCTG	TTGGCCCCCTTCGCCTGGGTCTGGTTTAAGAGC	TTAGCTCTTAAACCAGACCCAGGCGAAGGGGGCCAACAag
**HMNG1+34**	TACGGGACTCAGCCCGCGCC	TTGGTACGGGACTCAGCCCGCGCCGTTTAAGAGC	TTAGCTCTTAAACGGCGCGGGCTGAGTCCCGTACCAACAag

**IL17RA-20**	AGGCGCGACGGGCCAGGCCC	TTGGAGGCGCGACGGGCCAGGCCCGTTTAAGAGC	TTAGCTCTTAAACGGGCCTGGCCCGTCGCGCCTCCAACAag
**NTC**	GTAGCGAACGTGTCCGGCGT	TTGGTAGCGAACGTGTCCGGCGTGTTTAAGAGC	TTAGCTCTTAAACACGCCGGACACGTTCGCTACCAACAag

Supplementary Table 5. Primer sequences for RT-qPCR

**Name**	**Sequences (5'-3')**
**RT-q_GAPDH_Fw**	CAACAGCGACACCCACTCCT
**RT-q_GAPDH_Rev**	CACCCTGTTGCTGTAGCCAAA
**RT-q_HGMN1_Fw**	GCGAAGCCGAAAAAGGCAG
**RT-q_HGMN1_Rev**	TCCGCAGGTAAGTCTTCTTTAGT
**RT-q_IL17RA_1_Fw**	TCATCGTCTGCATGACCTGGAG
**RT-q_IL17RA_1_Rev**	GGCTGAGTAGATGATCCAGACC
**RT-q_KRAS_Fw**	GGACTGGGGAGGGCTTTCT
**RT-q_KRAS_Rev**	GCCTGTTTTGTGTCTACTGTTCT
**RT-q_MYC_general_Fw**	TGAGGAGACACCGCCCAC
**RT-q_MYC_general _Rev**	CAACATCGATTTCTTCCTCATCTTC
**RT-q_P1_MYC_Fw**	CTTGGCGGGAAAAAGAACGG
**RT-q_P1_MYC_Rev**	AGTTAGATAAAGCCCCGAAAACC
**RT-q _P2_MYC.Fw**	GTGGAAAACCAGCAGCCTCC
**RT-q _ P2_MYC.Rev**	TTGAGGGGCATCGTCGCG

Supplementary Table 6. CUT&Tag qPCR primers

Name	Sequences (5’-3’)
**C&T_q_MYC_Fw_CT**	GAGCTAGAGTGCTCGGCTG
**C&T_q_MYC Rev_CT**	GTGGGCGGAGATTAGCGAG
**C&T_q_MAZ _Fw_CT**	ACTCAGCGCAGGATTGTAAATA
**C&T_q _MAZ Rev_CT**	CCTCATGCTTCGGCTTCC
**C&T_q_RPA3 _Fw_CT**	CGGAAGTTGACAGATACAGGG
**C&T_q _RPA3 Rev_CT**	GATCGCAGAAAGGTAGTCTCAG
**C&T_q _RBBP4 _Fw_CT**	GAAAGCTACTCCGCGCGTCT
**C&T_q_RBBP4 Rev_CT**	ACCTTCGCGCCAACATCAG
**C&T_q_PVT1 _Fw_CT_2**	CTGCCGGGAAGCAGGCTG
**C&T_q_PVT1 _Rev_CT_2**	TGACGTCACGGCGGCTCG

Supplementary Table 7. Toxicity evaluation of Cl-PyPDS$_2$ and PyPDS in MCF7 WT and MCF7 cells stably expressing dCas9-Halo.

IC50 values were determined by a cell proliferation assay with ligand concentrations ranging from 0.25 to 10 μM. Data are presented as mean ± SEM from two independent biological replicates (n=2), each comprising three technical replicates. Concentration-response data were fitted with a variable-slope sigmoidal dose-normalised response (Hill) equation. Statistical differences between curves were evaluated using the extra-sum-of-squares F-test in GraphPad Prism 10.4, and the resulting F statistics, degrees of freedom, 95% confidence intervals and p-values are reported. The p-values were extracted from comparison of curve fit IC50: *** for PyPDS versus Cl-PyPDS2 in WT and **** in dCas9-Halo MCF7 cells. p-value: ns > 0.05, * ≤ 0.05, ** ≤ 0.01, *** ≤ 0.001, **** ≤ 0.0001.

Ligand	IC_{50} (μM) WT	F (DFn, DFd)	95% CI	p-value	IC_{50} (μM) dCas9-Halo	F (DFn, DFd)	95% CI	p-value
**PyPDS**	4.8 ± 0.5	14.25 (1, 24)	3.751 to 5.761	*** 0.0009	4.3 ± 0.4	20.43 (1, 24)	3.335 to 5.259	**** 0.0001
**PhenDC3**	>10	n/a	n/a	n/a	>10	n/a	n/a	n/a
**PDC**	>10	n/a	n/a	n/a	>10	n/a	n/a	n/a
**Cl-PyPDS$_2$**	7.3 ± 0.5	14.25 (1, 24)	6.279 to 8.352	*** 0.0009	6.9 ± 0.3	20.43 (1, 24)	6.164 to 7.661	**** 0.0001

**Cl-PhenDC3₂**	>10	n/a	n/a	n/a	>10	n/a	n/a	n/a
**Cl-PDC₂**	>10	n/a	n/a	n/a	>10	n/a	n/a	n/a
**Cl-pep-RVS₂**	>10	n/a	n/a	n/a	>10	n/a	n/a	n/a
**Cl-pep-RVS₄**	>10	n/a	n/a	n/a	>10	n/a	n/a	n/a
**DC-34**	>10	n/a	n/a	n/a	N/A	n/a	n/a	n/a

Supplementary Table 8. Summary of G4 targeted sequences, sgRNAs design, PAM sequences, and distance from the targeted G4.

**G4 targeted**	**DNA sequence of the predicted G4 (5'-3')**	**sgRNA sequence (5'-3')**	**PAM (5'-3')**	**Distance from the predicted G4 (bp)**
MYC-G4	TGGGGAGGGTGGGGAGGGTGGGGAAGG	AGCTAGAGTG CTCGGCTGCC	CGG	19
PVT1-G4	GCACCGGGCGGCGGGCGGGGACCTGGG GAAGGCCGGGAGCGC	GGCTCAGTGC CCTGCGCTGC	CGG	20
HMGN1-G4	GGGGTGGGGGCGCCCGGGGGGGGGGGG GGGGCCCCCGGAAGGGGGCGGGG	GCCCCCTTCG CCTGGGTCTG	GGG	22
IL17RA-G4	ACCTGGGGCGCGCGGGCGGAGGAGGGC CGGGGAGGGACGCCGGAAGGAC	AGGCGCGACG GGCCAGGCCC	AGG	20

Supplementary Table 9. G4-ligands-mediated thermal stabilization of the G4-forming hTelo oligonucleotide *via* FRET-melting.

ΔT_m values were determined by FRET-melting assays using 4 µM G4 ligand and 0.4 µM dual-labelled hTelo oligonucleotide. Data represent the mean of three independent experiments (n = 3). Statistical significance was calculated using a two-tailed t-test in GraphPad Prism. p-value: ns > 0.05, * ≤0.05, ** ≤0.01, *** ≤0.001, **** ≤0.0001.

**G4 Ligand (4 µM)**	**ΔT_m-hTelo ($T_{m4\ \mu M}$ - $T_{m\ 0\ \mu M}$)**	**p-value (paired, Two-tailed)**
PyPDS	16.48	*** (*P*= 0.0005)
Cl-PyPDS₂	N/A	* (*P*= 0.0370)
Cl-PyPDS₄	17	* (*P*= 0.0140)
PhenDC3	35.01	** (*P*= 0.0037)
Cl-PhenDC3₂	23.43	** (*P*= 0.0084)

Cl-PhenDC3$_4$	28.93	****(P= 0.0005)

Supplementary Table 10. G4-ligands-mediated thermal stabilization of the G4-forming BCL2 oligonucleotide *via* FRET-melting.

ΔT_m values were determined by FRET-melting assays using 4 µM G4 ligand and 0.4 µM dual-labelled BCL2 oligonucleotide. Data represent the mean of three independent experiments (n = 3). Statistical significance was calculated using a two-tailed t-test in GraphPad Prism. p-value: ns > 0.05, * ≤0.05, ** ≤0.01, *** ≤0.001, **** ≤0.0001.

G4 Ligand (4 µM)	ΔT_m-BCL2 ($T_{m4\ \mu M}$ - $T_{m\ 0\ \mu M}$)	p-value (paired, Two-tailed)
PyPDS	4.66	* (P=0.0225)
Cl-PyPDS$_2$	4.47	ns (P=0.3336)
Cl-PyPDS$_4$	5.08	* (P=0.0231)
PhenDC3	30.64	** (P=0.0011)
Cl-PhenDC3$_2$	25.58	** (P=0.0016)
Cl-PhenDC3$_4$	29.24	** (P=0.0023)

Supplementary Table 11. G4-ligands-mediated thermal stabilization of the G4-forming c-KIT2 oligonucleotide *via* FRET-melting.

ΔT_m values were determined by FRET-melting assays using 4 µM G4 ligand and 0.4 µM dual-labelled c-KIT2 oligonucleotide. Data represent the mean of three independent experiments (n = 3). Statistical significance was calculated using a two-tailed t-test in GraphPad Prism. p-value: ns > 0.05, * ≤0.05, ** ≤0.01, *** ≤0.001, **** ≤0.0001.

G4 Ligand (4 µM)	ΔT_m c-KIT2 ($T_{m4\,\mu M}$ - $T_{m\,0\,\mu M}$)	p-value (paired, Two-tailed)
PyPDS	14.93	** (*P*= 0.0003)
Cl-PyPDS$_2$	16.4	** (*P*=0.0090)
Cl-PyPDS$_4$	11.18	** (*P*=0.0021)
PhenDC3	20.91	*** (*P*=0.0004)
Cl-PhenDC3$_2$	13.71	** (*P*=0.0045)
Cl-PhenDC3$_4$	12.37	** (*P*=0.0023)

Supplementary Table 12. G4-ligands-mediated thermal stabilization of the G4-forming c-MYC-Pu22 oligonucleotide *via* CD-melting.

ΔT_m values were determined by CD-melting assays using increasing concentration (µM) of G4 ligand and 2 µM of c-MYC(Pu22) oligonucleotide. Data represent the mean of three independent experiments (n = 3). Statistical significance was calculated using a two-tailed t-test with Welch test correction in GraphPad Prism. p-value: ns > 0.05, * ≤0.05, ** ≤0.01, *** ≤0.001, **** ≤0.0001.

	Concentration (µM)							
	0.5		1.0		2.0		4.0	
**G4 Ligand**	ΔT_m	p-value	ΔT_m	p-value	ΔT_m	p-value	ΔT_m	p-value
**PyPDS**	6.4	**** 0.0001	12.3	**** < 0.0001	14.4	**** < 0.0001	17.6	**** < 0.0001
**Cl-PyPDS$_2$**	4.6	*** 0.0004	8.8	**** < 0.0001	11.2	**** < 0.0001	15.9	**** < 0.0001
**Cl-PyPDS$_4$**	-0.3	ns	7.3	**** < 0.0001	10.4	**** < 0.0001	13.1	**** < 0.0001
**PhenDC3**	11.0	** 0.0012	41.3	**** < 0.0001	59.5	ns (not accurate, no melting Tm>95 °C)	n/a	n/a

**Cl-PhenDC3$_2$**	9.6	** 0.0017	39.5	**** < 0.0001	57.1	ns (not accurate, no melting Tm>95 °C)	n/a	n/a
**Cl-PhenDC3$_4$**	6.0	** 0.0032	37.3	**** < 0.0001	44.8	ns (not accurate, no melting Tm>95 °C)	n/a	n/a

Supplementary Table 13. Cl-pep-RVS$_4$-mediated thermal stabilization of the i-motif-forming c-MYC-C52 oligonucleotide via CD-melting.

CD melting analysis of 10 µM c-MYC-C52 in 10 mM Sodium Cacodylate at pH 6.6 buffer in the presence of 10 molar equivalents of Pep-RVS or the DMSO solvent control. All data are presented as Mean change in the higher Tm ± SEM (n=2). Significance was tested with a one-way ANOVA with Bonferroni post-hoc analysis. p-value: ns > 0.05, * ≤0.05, ** ≤0.01, *** ≤0.001, **** ≤0.0001.

Melting Temperature (°C)	DMSO	Cl-pep-RVS$_4$
c-MYC-C52	33 ± 0.6	39 ± 0.2** (P= 0.0027)
	83 ± 0.3	

Supplementary Table 14. Summary table of mRNA-seq results.

Differentially expressed genes were identified for the category reported using DESeq2 with the following parameters (FDR < 0.05) and log2 fold-change thresholds (|log2FC| ≥ 1).

**Category**	**ATENA (sgRNA$_{MYC\text{-}19}$) vs mock**	**ATENA (sgRNA$_{MYC\text{-}19}$) vs mock NTC-filtered**	**ATENA (sgRNA NTC) vs mock**	**DC-34 vs mock**	**PyPDS vs mock**
**Upregulated**	112	33	122	81	1907
**Downregulated**	18	10	23	39	319

Supplementary Table 15. Summary table of shared DEGs between ATENA and PyPDS.

Differentially expressed genes were identified for the category reported using DESeq2 with the following parameters (FDR < 0.05) and log2 fold-change thresholds (|log2FC| ≥ 1).

**Category**	**PyPDS upregulated**	**PyPDS downregulated**
**ATENA upregulated**	2(ALPK3, CCN1)	3 (EPPK1, SLC38A2, INSR)

**ATENA downregulated**	1(DLX1)	N/A

Supplementary Table 16. Summary table of shared DEGs between ATENA and DC-34.

Differentially expressed genes were identified for the category reported using DESeq2 with the following parameters (FDR < 0.05) and log2 fold-change thresholds (|log2FC| ≥ 1).

**Category**	**DC-34 upregulated**	**DC-34 downregulated**
**ATENA upregulated**	3(NEU1, ATF3,TACC2)	N/A
**ATENA downregulated**	N/A	N/A

Supplementary Table 17. Summary table of shared DEGs between PyPDS and DC-34.

Differentially expressed genes were identified for the category reported using DESeq2 with the following parameters (FDR < 0.05) and log2 fold-change thresholds (|log2FC| ≥ 1).

**Category**	**DC-34 upregulated**	**DC-34 downregulated**
**PyPDS upregulated**	12(LEKR1,CALHM1,MT-RNR1, IFIT2,HCLS1,RGPD5,MT-RNR2, CTRB2, RIPOR3, CYP1A1, CYP1B1-AS1, MT-TL1)	8 (UCKL1-AS1, ARRDC4,RSKR GRIN1,PROB1,KRT15,KCNQ1OT1 HSALR1)
**PyPDS downregulated**	3 (PRUNE2, SLC7A11,SLITRK6)	1 (AHNAK2)

Supplementary Table 18. Comparative overview of single-G4 targeting platforms.

**Tool**	**Advantages**	**Limitation**
ATENA	Modular platform (can be easily adapted to target any DNA structure of interest using the same design principles);<br>chemical synthesis accessible with modular and derivatizable scaffold production;	PAM-constrains in the target region; steric block possible if sgRNA overlaps core promoter regions.

	reduced steric occupancy of the fusion proteins (dCas9-Halo);<br>single-G4s and iMs precision; minimal promoter occlusion when sgRNAs is avoided in the –50/+300 bp window.	
Qin, G. et al. Targeting specific DNA G-quadruplexes with CRISPR-guided G-quadruplex-binding proteins and ligands. Nat Cell Biol 26, 1212-1224 (2024) [34].	Protein and ligand mediated single-G4s stabilisation.	PAM-constrains in the target region; large fusion protein (dCas9-nucleolin) which might results in crowding effects at the target promoters with consequent road-block effects which impact transcription in a non-G4 related manner; ligand library synthetic approach less flexible; Use of biotin-mSA approach which can be imprecise and may lead to non-specific binding.
Berner, A. *et al.* G4-Ligand-Conjugated Oligonucleotides Mediate Selective Binding and Stabilization of Individual G4 DNA Structures. *J Am Chem Soc* **146**, 6926-6935 (2024) [61].	Fully synthetic; no protein; nanomolar affinity; easy to swap guide.	Requires single-stranded access for hybridisation; oligo delivery *in vivo*; chemical synthesis for each target.<br>Usage of multiple sgRNAs on a single target.
Tan, D. J. Y. *et al.* Guanine anchoring: a strategy for specific targeting of a G-quadruplex using short PNA, LNA and DNA molecules. Chem Commun (Camb) 56, 5897-5900 (2020)[63].	small size; nuclease-stable chemistry possible.	cell uptake of PNAs remains challenging.
He, Y. D. *et al.* Selective Targeting of Guanine-Vacancy-Bearing G-Quadruplexes by G-	Picomolar selectivity.	Take only advantage of the G-vacancy-bearing G-quadruplexes (GVBQs).

Quartet Complementation and Stabilization with a Guanine-Peptide Conjugate. J Am Chem Soc 142, 11394-11403 (2020)[62].		
Zhao, H., *et al.* Selective recognition of RNA G-quadruplex in vitro and in cells by L-aptamer-D-oligonucleotide conjugate. Nucleic Acids Res 52, 13544-13560 (2024)[64].	Demonstrate a transcriptional control via targeting rG4-containing transcripts in cells.	Only focused on RNA G4 and DNA G4 is not address.

Supplementary-4 Supplementary Figures

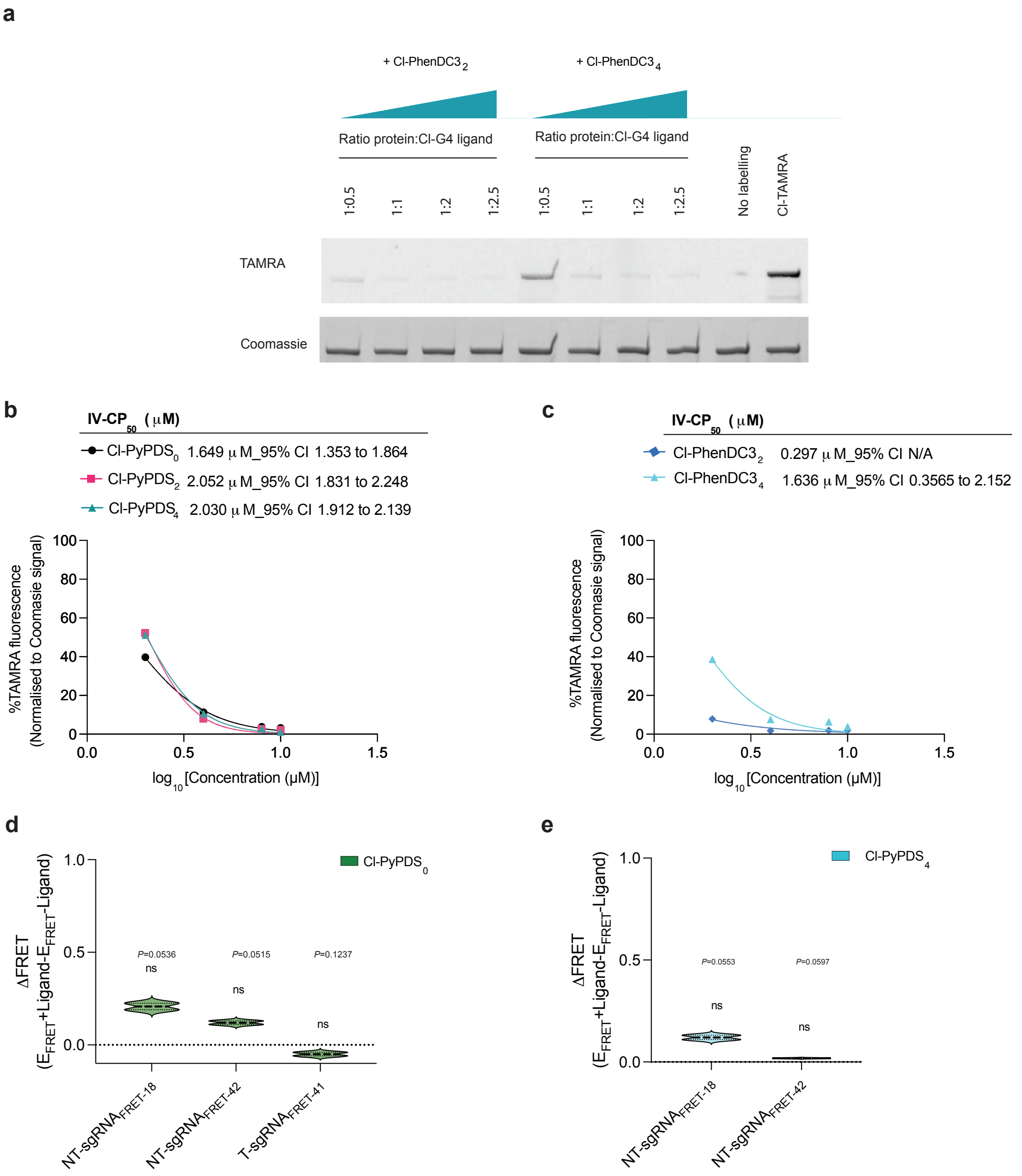

Supplementary Figure 3 ATENA biochemical characterization a, SDS-Page gel of the Cl-PhenDC3$_n$ competition assay showing each sample's fluorescent level acquired in the TAMRA channel (542 nm) and the corresponding protein level (Coomassie staining). (n=2) **b, c,** Plot of the band intensity value in the TAMRA channel indicative of dCas9-Halo labeling efficiency *in vitro* for Cl-PyPDS$_2$ and Cl-PhenDC3$_2$, respectively, and relative IV-CP$_{50}$. Each band intensity was quantified using Image Studio software and normalized for the corresponding Coomassie signal, expressed as a percentage (with 100% labeling corresponding to the positive control). IV-CP$_{50}$ values were determined using nonlinear regression (dose-response inhibition curves with constrained fitting) in GraphPad Prism (n = 2) with R^2 values of 0.9957 for Cl-PyPDS$_0$, R^2 values of 0.9956 for Cl-PyPDS$_2$ and R^2 values of 0.9988 for Cl-PyPDS$_4$. R^2 values N/A for Cl-PhenDC3$_2$ and 0.9582 for Cl-PhenDC3$_4$. **d, e** ΔFRET efficiency of the decorated dCas9-PDSx(with Cl-

$PyPDS_0$ and Cl-$PyPDS_4$, respectively) targeting c-KIT2-G4. The values indicated were extrapolated from the band intensity measured in the Cy3 and Cy5 channels (Typhoon FLA 9500). The signals in both channels were normalised for the background and the sgRNA NTC control. The normalised fluorescence values were then used to calculate_the ΔFRET efficiency as follows for each sgRNA: FRET-Efficiency(E)+$ligand_{sgRNAx}$ – FRET-Efficiency(E)-$ligand_{sgRNAx}$ (n=2). The data presented are the mean of n = number of independent experiments. Statistical significance was calculated using a Welch-corrected two-tailed t-test in GraphPad Prism;_p-value: ns > 0.05, * ≤0.05, ** ≤0.01, *** ≤0.001, **** ≤0.0001. Source data are provided as a Source Data file.

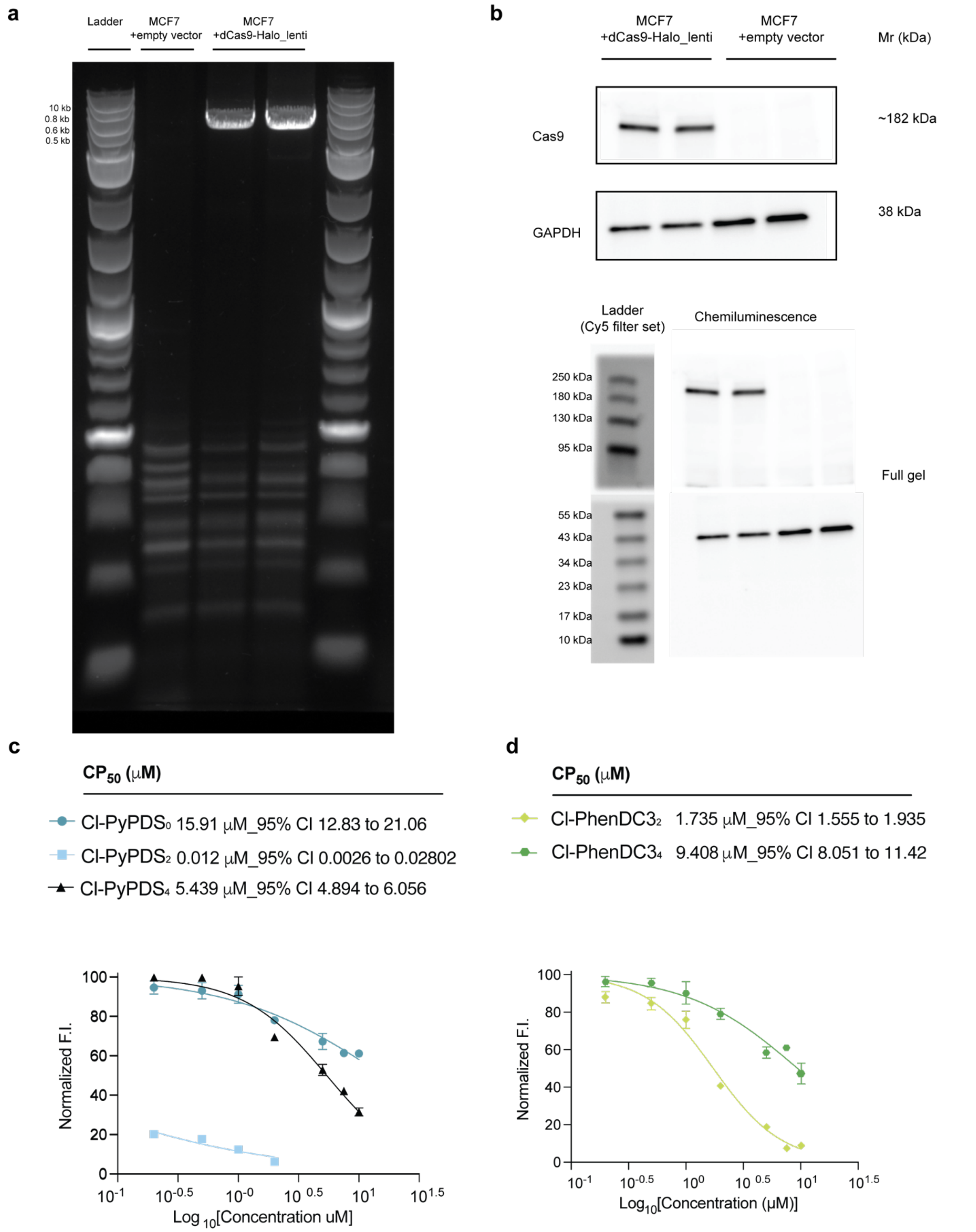

Supplementary Figure 4 ATENA optimization in mammalian cells. a, Genotyping of MC7 cells transduced with either empty vector or dCas9-Halo lentiviral construct showing the amplified expected band at 5.1 kb (SC_dCas9genotyping.Fw, SC_dCas9genotyping.Rev) in case of successful integration. **b,**

Western blot of either MCF7+dCas9-Halo stable cell line or MCF7+empty vector to confirm protein expression of dCas9-Halo. Gel images were acquired using Image Quant LAS 4000 (Cytiva) following the methodology described in the Methods section. **c, d,** Labeling efficiency of dCas9–Halo in live cells treated with Cl-PyPDS$_n$ or Cl-PhenDC3$_n$ and the corresponding CP$_{50}$ values. Cells labeled with the Cl-OG fluorophore were analyzed by flow cytometry (see Methods), and data were processed in FlowJo. Mean fluorescence values from 3 biological replicates (each with three technical replicates) were normalized to the positive-control signal and expressed as percent labeling. CP$_{50}$ values were obtained by nonlinear regression (dose–response inhibition curves with constrained fitting) in GraphPad Prism (Mean ± SD , n = 3). The fits yielded R^2 values of 0.9374 for Cl-PyPDS$_0$, 0.8598 for Cl-PyPDS$_2$, 0.9686 for Cl-PyPDS$_4$, 0.9775 for Cl-PhenDC3$_2$, and 0.9406 for Cl-PhenDC3$_4$. Data points at concentrations ≥5 μM were excluded from the Cl-PyPDS$_2$ fit because the compound was cytotoxic at those levels, as confirmed by its IC$_{50}$ values calculated (see Supplementary Table 7). Source data are provided as a Source Data file.

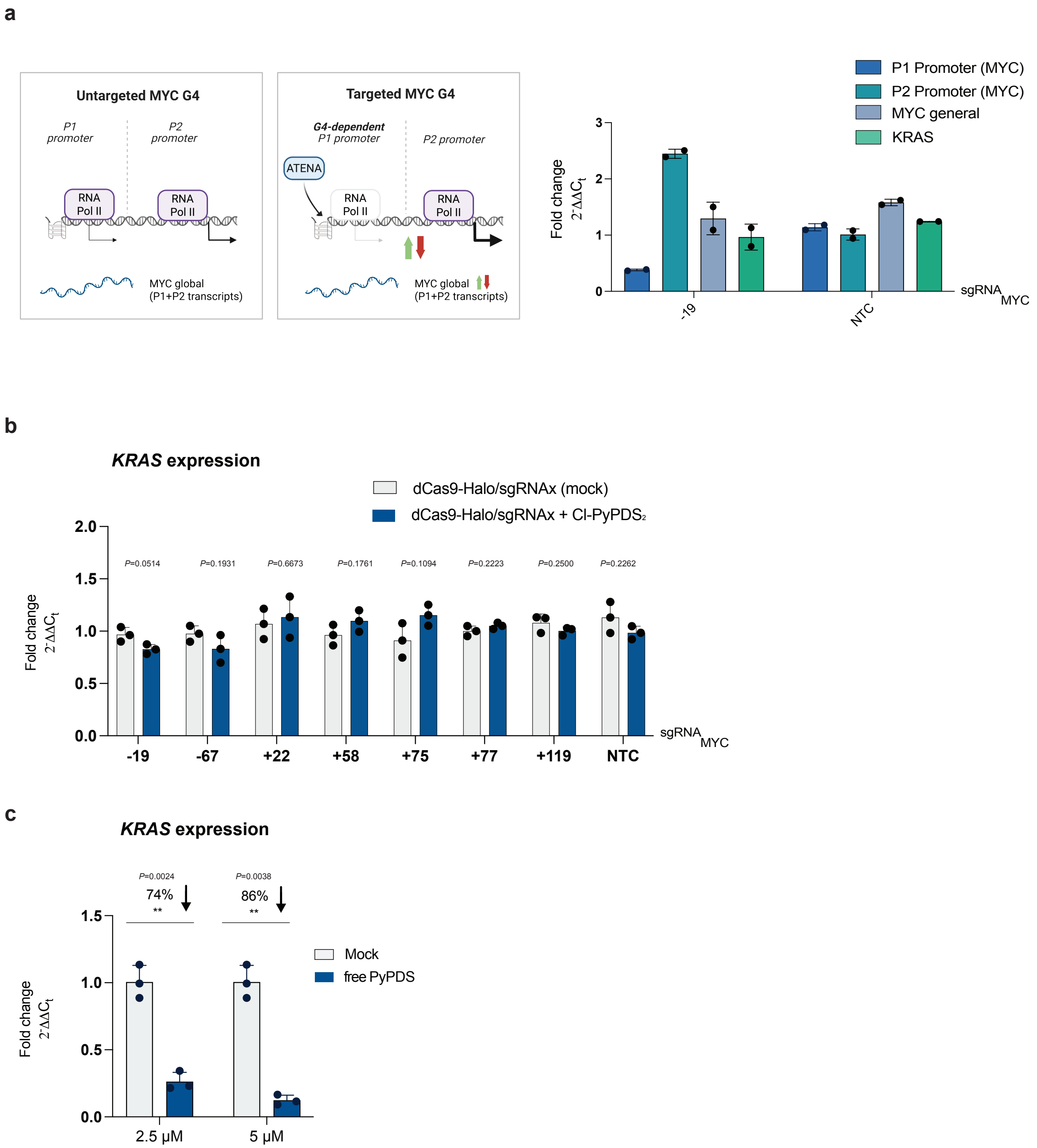

Supplementary Figure 5 P2-compensation and limited off-target activity following ATENA-mediated MYC-G4 targeting. a, (left) proposed model in which P2-driven transcription compensates for the reduction of P1-mediated transcription that follows MYC-G4 targeting (created with BioRender, https://BioRender.com/05eatvp). (right) RT-qPCR of the indicated genes in MCF7 cells stably expressing dCas9-Halo transfected with sgRNAMYC-19 or sgRNA NTC and incubated for 48h in the presence of 2.5 µM of Cl-PyPDS2 or DMSO (mock). The expression values are represented as fold change (2-ΔΔCt) with respect to the mock (DMSO-treated) transfected samples and after normalization for the housekeeping gene (GAPDH). n=2, biological replicates, each with three technical replicates. **b,** RT-

qPCR for KRAS expression in MCF7 cells stably expressing dCas9-Halo transfected with the indicated sgRNAs and incubated for 48h in the presence of (2.5 μM) Cl-PyPDS2. The expression values are represented as fold change (2-ΔΔCt) with respect to the mock (DMSO-treated) and normalized for the housekeeping gene (GAPDH). Mean ± SD, n=3, biological replicates, each with three technical replicates.**c**, RT-qPCR for KRAS expression in MCF7 cells incubated for 24h in the presence of (2.5 μM) free PyPDS. The expression values are represented as fold change (2-ΔΔCt) with respect to the mock (DMSO-treated) and after normalization for the housekeeping gene (GAPDH). Mean ± SD, n=3, biological replicates, each with three technical replicates. The data presented are the mean of n = number of independent biological samples. Statistical significance was calculated using a Welch-corrected two-tailed t-test in GraphPad Prism; p-value: ns > 0.05, * ≤0.05, ** ≤0.01, *** ≤0.001, **** ≤0.0001. Source data are provided as a Source Data file.

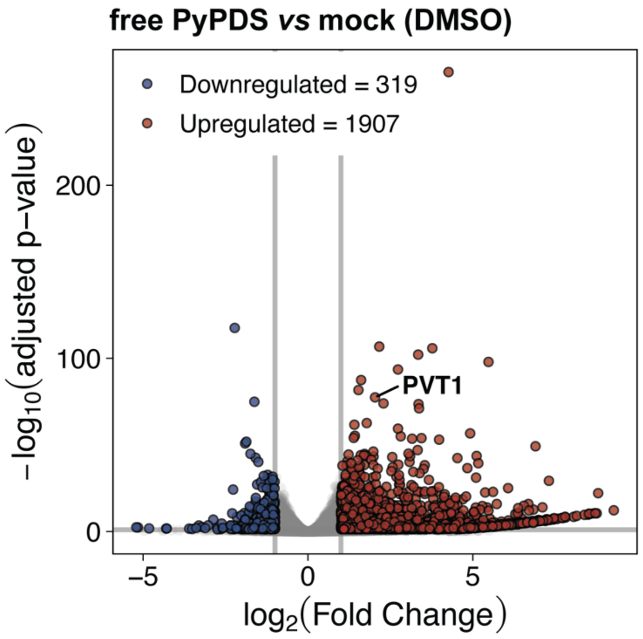

Supplementary Figure 6 Investigating free PyPDS effect on global mRNA expression in MCF7 cells. Volcano plot showing the number of genes differently expressed upon treatment of MCF7 cells with (2.5 uM) free PyPDS. The plot was generated by comparing Cl-PyPDS$_2$-treated samples and DMSO-treated (mock) samples using DESeq2 with FDR=0.05. Highlighted the *PVT1* gene, whose expression was previously shown to be upregulated after PDS treatment [35].

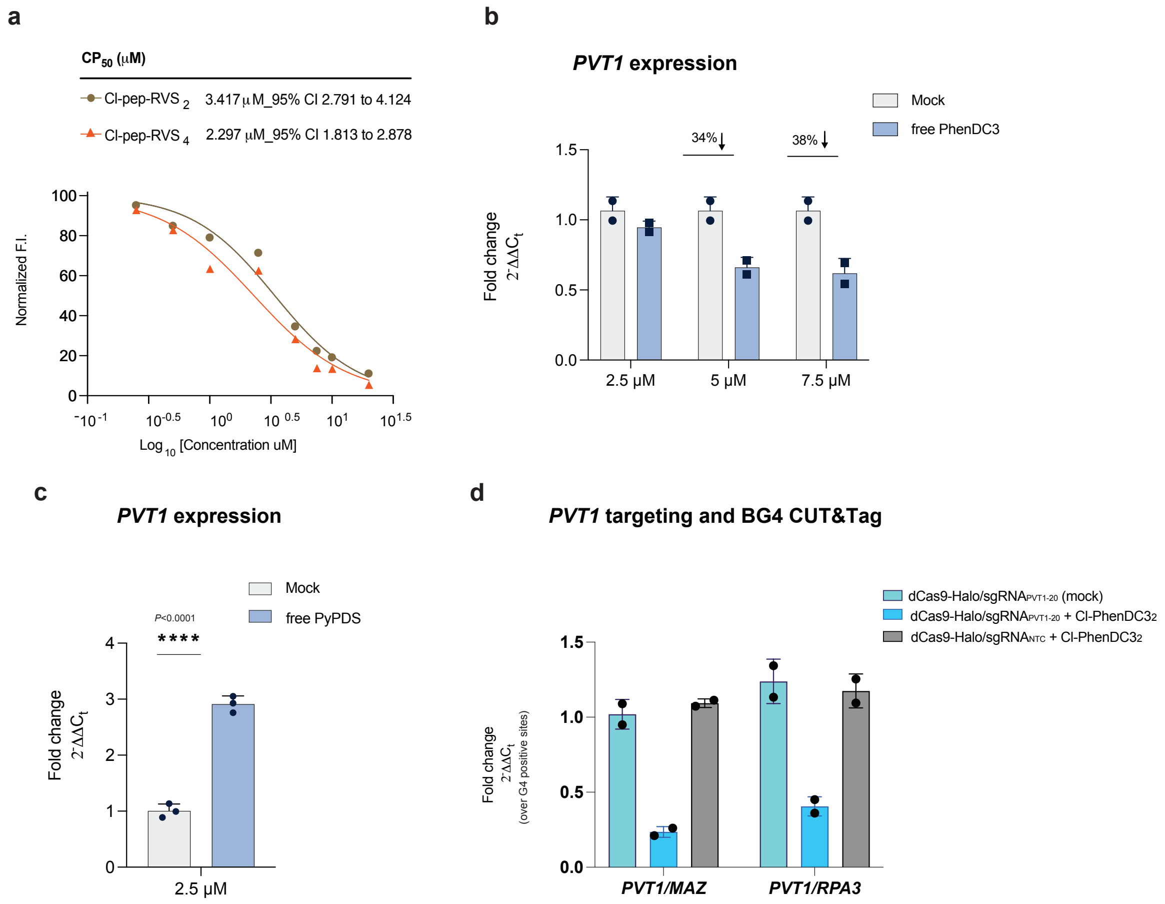

Supplementary Figure 7 ATENA targets the *c-MYC* i-motif and *lncPVT1*. a, Labeling efficiency of dCas9–Halo in live cells treated with Cl-pep-RVS$_2$ or Cl-pep-RVS$_4$ and the corresponding CP$_{50}$ values. Cells labelled with the Cl-OG fluorophore were analyzed by flow cytometry (see Methods), and data were processed in FlowJo. Mean fluorescence values from two biological replicates (each with three technical replicates) were normalized to the positive-control signal and expressed as percent labeling. CP$_{50}$ values were obtained by nonlinear regression (dose–response inhibition curves with constrained fitting) in GraphPad Prism (n = 2). The fits yielded R^2 values of 0.9548 for Cl-pep-RVS$_2$ and 0.9491 for Cl-pep-RVS$_4$. **b,** RT-qPCR for *PVT1* expression in MCF7 cells treated with increasing concentration of free PhenDC3 for 24h or DMSO (mock). The expression values are represented as fold change ($2^{-\Delta\Delta Ct}$) with respect to the mock (DMSO-treated) transfected samples and after normalization for the housekeeping gene *(GAPDH*). n=2, biological replicates, each with three technical replicates. **c**, RT-qPCR for *PVT1* expression in MCF7 cells treated with (2.5 µM) free PyPDS for 24h or DMSO (mock). The expression values are represented as fold change ($2^{-\Delta\Delta Ct}$) with respect to the mock (DMSO-treated) transfected samples and after normalization for the housekeeping gene *(GAPDH*). Mean ± SD, n=3, biological replicates, each with three technical replicates. **d,** BG4 CUT&Tag-qPCR for MCF7 cells stably expressing dCas9-Halo transfected with either sgRNA$_{PVT1-20}$ or sgRNA NTC and treated with DMSO (mock) or (2.5 µM) Cl-PhenDC3$_2$. BG4 accessibility was analyzed for *PVT1* and normalized to two G4s in control gene sites (*MAZ* and *RPA3*). n=2, biological replicates each with three technical replicates for

BG4 and one for the negative (no BG4 treatment). Data presented are mean of n = number of independent biological samples. Statistical significance was calculated using a Welch-corrected two-tailed t-test in GraphPad Prism; p-value: ns > 0.05, * ≤0.05, ** ≤0.01, *** ≤0.001, **** ≤0.0001. Source data are provided as a Source Data file.

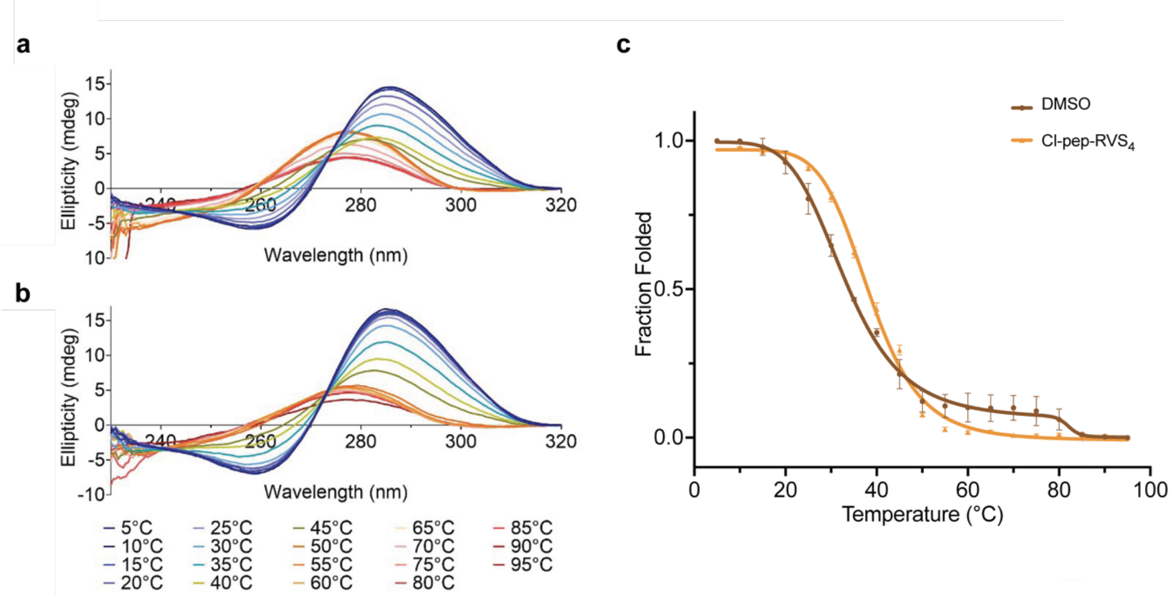

Supplementary Figure 8 CD melting curves of Cl-pep-RVS$_4$. 10 µM c-MYC-C52 oligonucleotide in the presence of 10 molar equivalents of DMSO (a) or Cl-pep-RVS$_4$ (b) and the relative normalised fraction folded (c). (n=2). Data presented are mean of n = number of independent experiments.

a

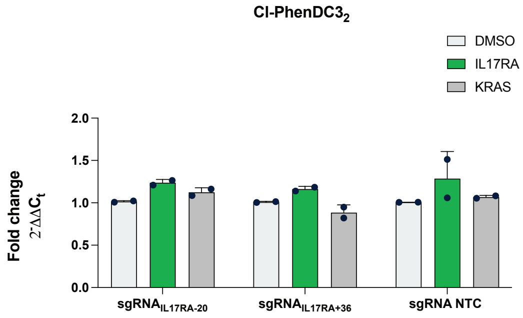

b

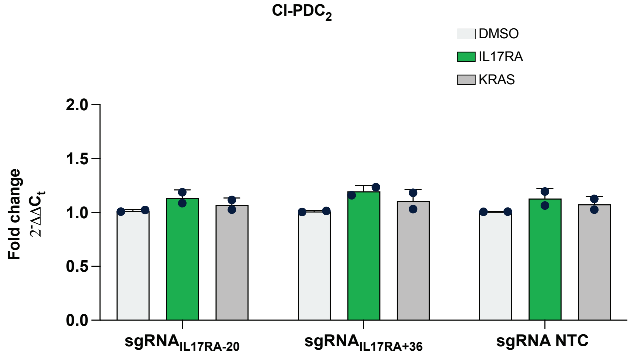

Supplementary Figure 9 *IL17RA* expression is not affected when targeting the G4 located in its promoter. RT-qPCR for IL17RA expression in MCF7 cells stably expressing dCas9-Halo, transfected with either $sgRNA_{IL17RA-20}$, $sgRNA_{IL17RA+36}$ or sgRNA NTC and treated with (2.5 µM) Cl-PhenDC3_2 (a) or Cl-PDC$_2$ (b) or 48h after transfection. The expression values are represented as fold change (2-ΔΔCt) with respect to the mock (DMSO-treated) and normalized for the housekeeping gene GAPDH; n=2, biological replicates, each of which includes three technical replicates. Data presented are the mean of n = number of independent biological samples. Statistical significance was calculated using a Welch-corrected two-tailed t-test in GraphPad Prism; p-value: ns > 0.05, * ≤0.05, ** ≤0.01, *** ≤0.001, **** ≤0.0001. Source data are provided as a Source Data file.

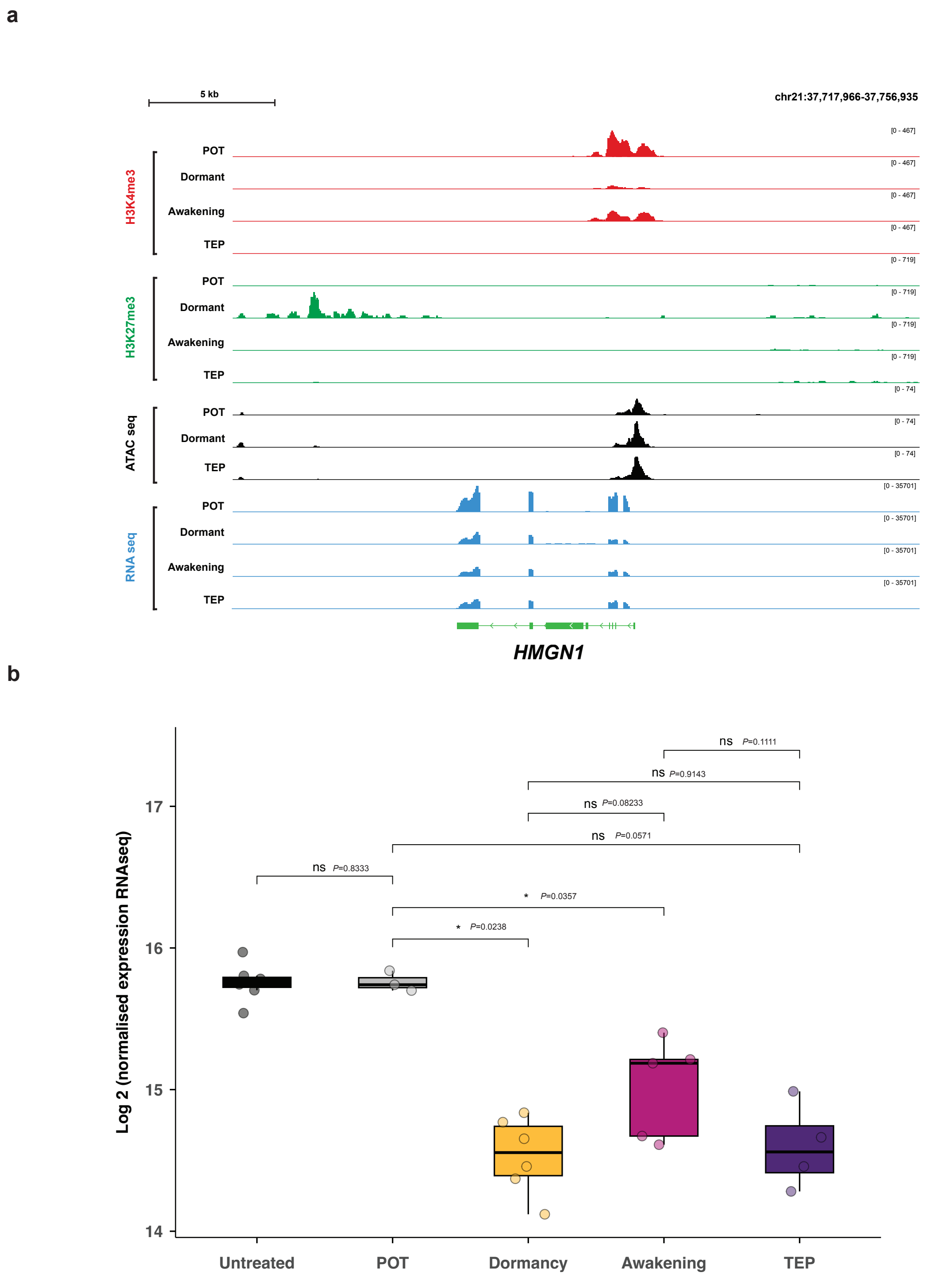

Supplementary Figure 10 Epigenetic and transcriptional landscape at the *HMGN1* locus in MCF7 cells under different Estrogen-deprivation conditions. a, Genome browser tracks display group-auto-

scaled CUT&Tag signal intensity for the histone mark H3K4me3 (red), and H3K27me3 (green); ATAC-seq signal intensity (black) and RNA-seq coverage (blue) across the *HMGN1* locus. Each track corresponds to a distinct time point in Estrogen-deprivation treatment (POT (Day 0), Dormancy (Day 43), Awakening, and TEP - Terminal End Point, as indicated on the left. Peaks in the H3K4me3 and RNA-seq tracks are associated with active promoters and transcriptional activity, respectively, while signal in the H3K27me3 track indicates repressive chromatin. Gene models are shown at the bottom, with exon-intron structure and transcriptional direction indicated. **b,** Boxplots showing gene expression levels of *HMGN1* gene as log-transformed DESeq2 and normalized counts from bulk RNAseq in MCF7 cells subjected to Estrogen deprivation treatment (from the depository GSE234171[5]). Untreated: MCF7 grown in complete media with Estradiol, POT: Starting population of MCF7 grown in complete media with Estradiol. Dormancy: MCF7 cells were grown in estrogen-deprived medium for 1-3 months. Awakening: Dormant populations that resumed proliferation. TEP (Terminal End Points): awakening populations subcultured for at least 30 days. Statistical significance was determined using the Wilcoxon rank-sum test, with p-values indicated by asterisks (*), n=6.

BD FACSDiva 9.4

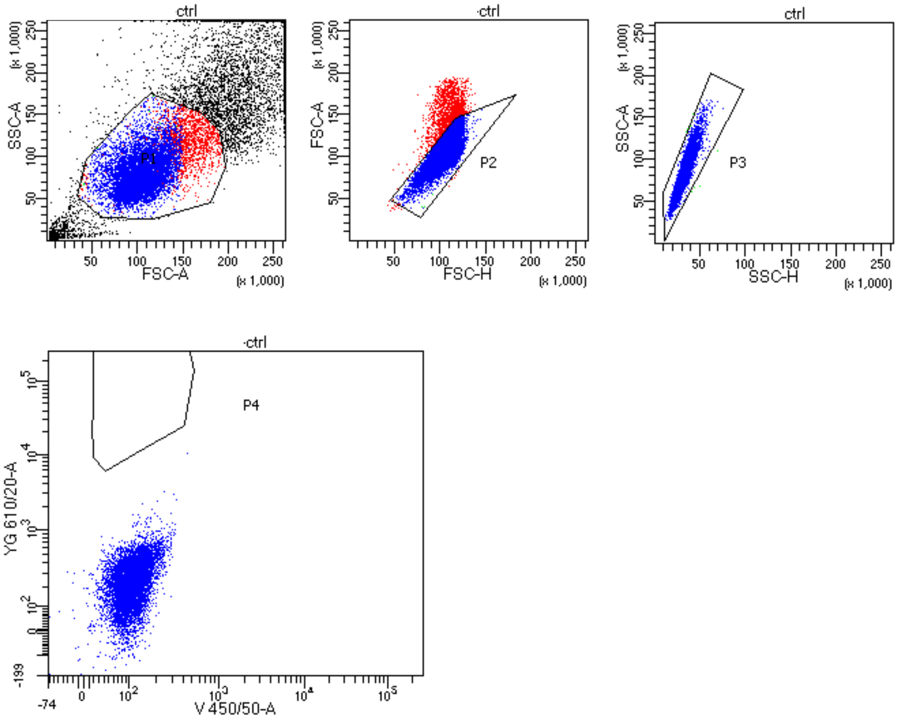

Tube: ctrl

Population	#Events	%Parent	%Total
All Events	16,140	####	100.0
P1	9,930	61.5	61.5
P2	8,284	83.4	51.3
P3	8,274	99.9	51.3
P4	0	0.0	0.0

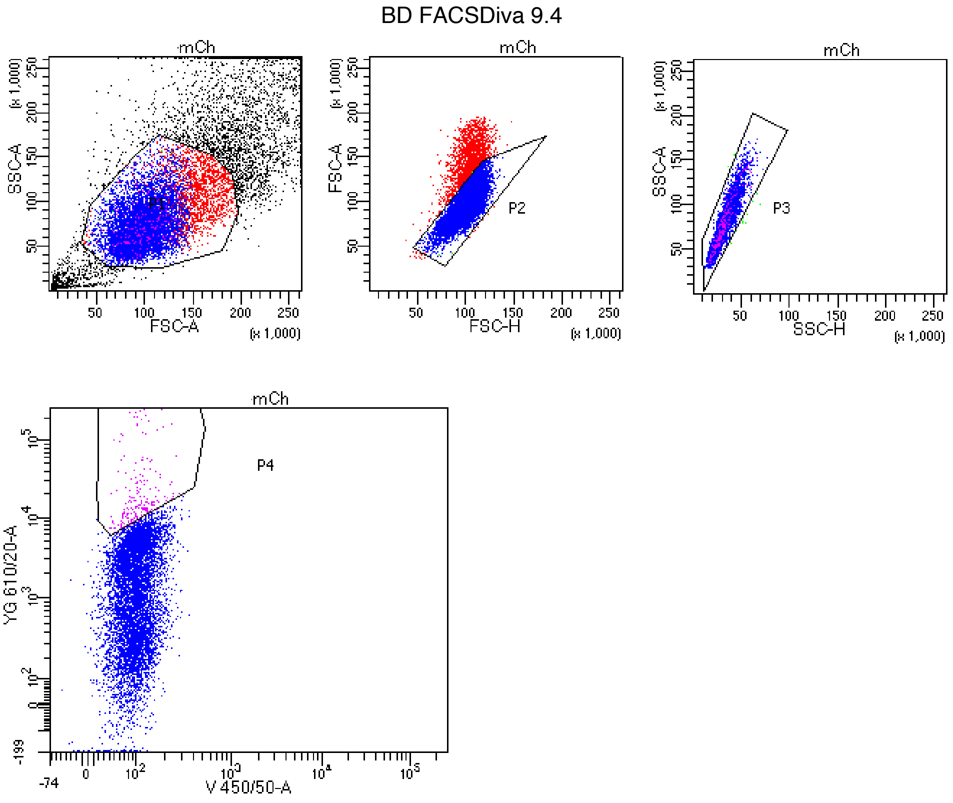

Tube: mCh

Population	#Events	%Parent	%Total
All Events	14,636	####	100.0
P1	9,976	68.2	68.2
P2	8,012	80.3	54.7
P3	7,997	99.8	54.6
P4	217	2.7	1.5

Supplementary Figure 11 FACS sorting of single Lenti-dCas9Halo-T2A-mCherry transduced clones. Plots of gating strategy for sorting mCherry-positive transduced cells with Lenti-dCas9Halo-T2A-mCherry construct. On the left, gating is based on untransduced cells, and on the right, gating of the transduced cells for subsequent single-cell sorting.

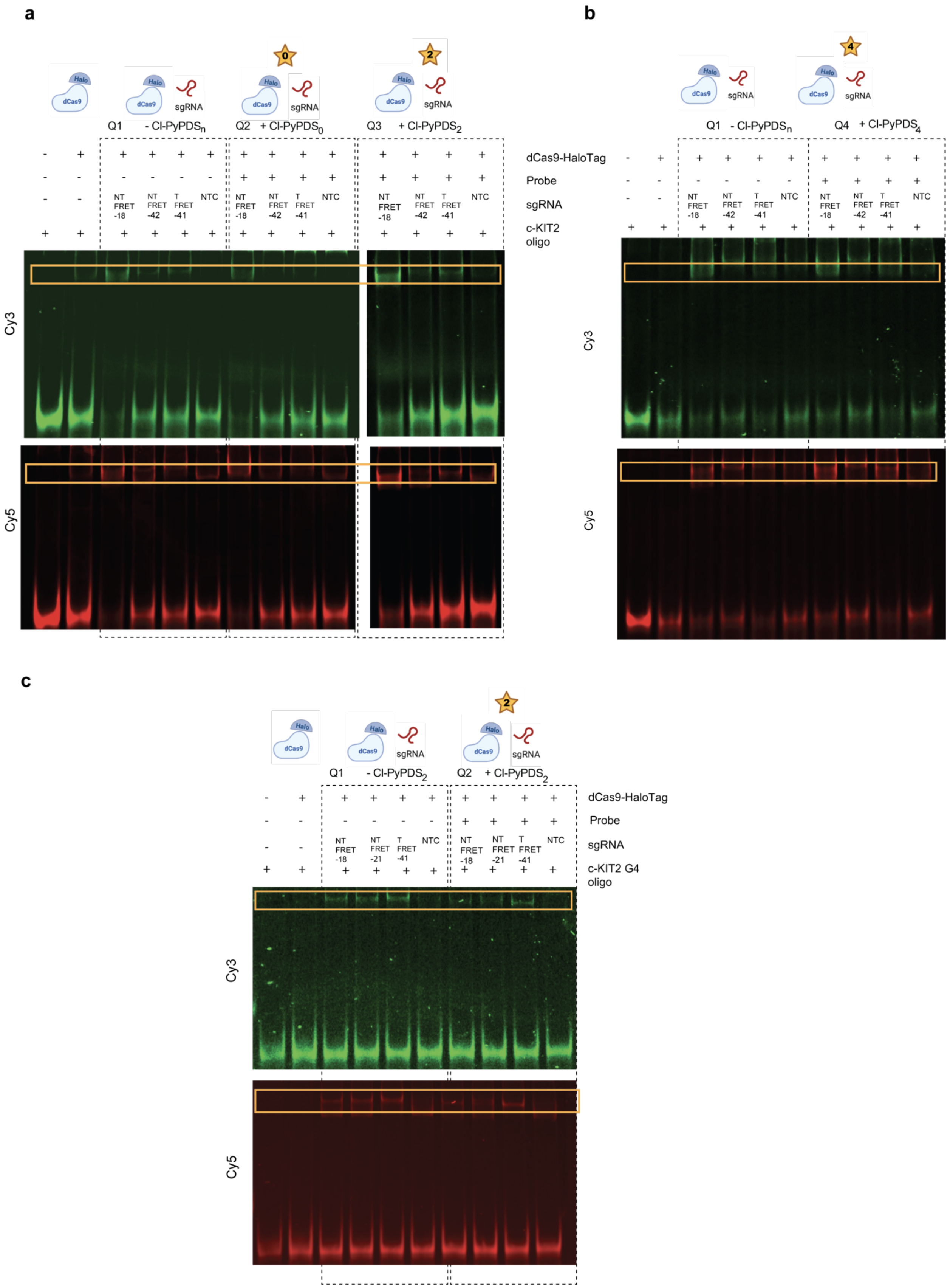
a
b
c
Halo
dCas9
sgRNA
Q1 - Cl-PyPDSn
Q2 + Cl-PyPDS0
Q3 + Cl-PyPDS2
Q4 + Cl-PyPDS4
Q1 - Cl-PyPDS2
Q2 + Cl-PyPDS2
dCas9-HaloTag
Probe
sgRNA
c-KIT2 oligo
c-KIT2 G4 oligo
NT FRET -18
NT FRET -42
NT FRET -21
T FRET -41
NTC
Cy3
Cy5

Supplementary Figure 12 EMSA gel-based FRET assay of dCas9-HaloTag/Cl-PyPDS$_n$ complex bound to c-KIT2 oligo.

a, Q1: Control conditions with dCas9-HaloTag without prior incubation with Cl-PyPDS$_n$ probes and the indicated sgRNAs; Q2-Q3: Experimental conditions with the Cl-PyPDS$_0$ and Cl-PyPDS$_2$ probe respectively and the indicated sgRNAs; **b,** Q1: Control conditions with dCas9-HaloTag without Cl-G4n probe Q4: Experimental conditions with the Cl-PyPDS$_4$ probe and the indicated sgRNAs. **c,** Q1: Control conditions with dCas9-HaloTag without prior incubation with Cl-PyPDS$_n$ probes and the indicated sgRNAs; Q2: Experimental conditions with the Cl-PyPDS$_2$ probe and the indicated sgRNAs. The gels were acquired in both the Cy3 (575 nm emission filter) and Cy5 (665 nm emission filter) fluorescence channels with Typhoon FLA 9500. Yellow squares correspond to the identification and quantification of the shifted gel bands.

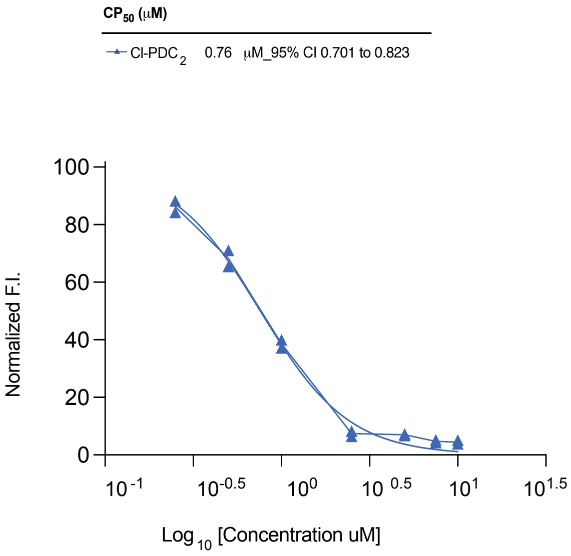

Supplementary Figure 13 CAPA assay for Cl-PDC$_2$ ligand. Labeling efficiency of dCas9–Halo in live cells treated with Cl-PDC$_2$, the corresponding CP_{50} value. Cells labeled with the Cl-OG fluorophore were analyzed by flow cytometry (see Methods), and data were processed in FlowJo. Mean fluorescence values from two biological replicates (each with three technical replicates) were normalized to the positive-control signal and expressed as percent labeling. CP_{50} values were obtained by nonlinear regression (dose–response inhibition curves with constrained fitting) in GraphPad Prism (n = 2). The fits yielded an R^2 value of 0.9911.

Supplementary-5 Supplementary plasmid sequence: Lentiviral-dCas9-Halo

gtcgacggatcgggagatctcccgatcccctatggtgcactctcagtacaatctgctctgatgccgcatagttaagccagtatctgctccctgcttgtgtgttggag
gtcgctgagtagtgcgcgagcaaaatttaagctacaacaaggcaaggcttgaccgacaattgcatgaagaatctgcttagggttaggcgttttgcgctgcttcgcg
atgtacgggccagatatacgcgttgacattgattattgactagttattaatagtaatcaattacggggtcattagttcatagcccatatatggagttccgcgttacataa
cttacggtaaatggcccgcctggctgaccgcccaacgacccccgcccattgacgtcaataatgacgtatgttcccatagtaacgccaatagggactttccattgac
gtcaatgggtggagtatttacggtaaactgcccacttggcagtacatcaagtgtatcatatgccaagtacgccccctattgacgtcaatgacggtaaatggcccgc
ctggcattatgcccagtacatgaccttatgggactttcctacttggcagtacatctacgtattagtcatcgctattaccatggtgatgcggttttggcagtacatcaatg

ggcgtggatagcggtttgactcacggggatttccaagtctccaccccattgacgtcaatgggagtttgttttggcaccaaaatcaacgggactttccaaaatgtcgt
aacaactccgccccattgacgcaaatgggcggtaggcgtgtacggtgggaggtctatataagcagcgcgttttgcctgtactgggtctctctggttagaccagatc
tgagcctgggagctctctggctaactagggaacccactgcttaagcctcaataaagcttgccttgagtgcttcaagtagtgtgtgcccgtctgttgtgtgactctggt
aactagagatccctcagacccttttagtcagtgtggaaaatctctagcagtggcgcccgaacagggacttgaaagcgaaagggaaaccagaggagctctctcg
acgcaggactcggcttgctgaagcgcgcacggcaagaggcgaggggcggcgactggtgagtacgccaaaaattttgactagcggaggctagaaggagaga
gatgggtgcgagagcgtcagtattaagcgggggagaattagatcgcgatgggaaaaaattcggttaaggccagggggaaagaaaaaatataaattaaaacata
tagtatgggcaagcagggagctagaacgattcgcagttaatcctggcctgttagaaacatcagaaggctgtagacaaatactgggacagctacaaccatcccttc
agacaggatcagaagaacttagatcattatataatacagtagcaaccctctattgtgtgcatcaaaggatagagataaaagacaccaaggaagctttagacaagat
agaggaagagcaaaacaaaagtaagaccaccgcacagcaagcggccgctgatcttcagacctggaggaggagatatgagggacaattggagaagtgaatta
tataaatataaagtagtaaaaattgaaccattaggagtagcacccaccaaggcaaagagaagagtggtgcagagagaaaaaagagcagtgggaataggagctt
tgttccttgggttcttgggagcagcaggaagcactatgggcgcagcgtcaatgacgctgacggtacaggccagacaattattgtctggtatagtgcagcagcaga
acaatttgctgagggctattgaggcgcaacagcatctgttgcaactcacagtctggggcatcaagcagctccaggcaagaatcctggctgtggaaagatacctaa
aggatcaacagctcctggggatttggggttgctctggaaaactcatttgcaccactgctgtgccttggaatgctagttggagtaataaatctctggaacagatttgga
atcacacgacctggatggagtgggacagagaaattaacaattacacaagcttaatacactccttaattgaagaatcgcaaaaccagcaagaaaagaatgaacaa
gaattattggaattagataaatgggcaagtttgtggaattggtttaacataacaaattggctgtggtatataaaattattcataatgatagtaggaggcttggtaggttta
agaatagtttttgctgtactttctatagtgaatagagttaggcagggatattcaccattatcgtttcagacccacctcccaaccccgaggggacccgacaggcccga
aggaatagaagaagaaggtggagagagagacagagacagatccattcgattagtgaacggatcggcactgcgtgcgccaattctgcagacaaatggcagtatt
catccacaattttaaaagaaaaggggggattggggggtacagtgcaggggaaagaatagtagacataatagcaacagacatacaaactaaagaattacaaaaa
caaattacaaaaattcaaaattttcgggtttattacagggacagcagagatccagtttggttaattagctagctgcaaagatggataaagttttaaacagagaggaat
ctttgcagctaatggaccttctaggtcttgaaaggagtgggaattggctccggtgcccgtcagtgggcagagcgcacatcgcccacagtccccgagaagttggg
gggaggggtcggcaattgaaccggtgcctagagaaggtggcgcggggtaaactgggaaagtgatgtcgtgtactggctccgcctttttcccgagggtggggg
agaaccgtatataagtgcagtagtcgccgtgaacgttctttttcgcaacgggtttgccgccagaacacaggtaagtgccgtgtgtggttcccgcgggcctggcctc
tttacgggttatggcccttgcgtgccttgaattacttccacctggctgcagtacgtgattcttgatcccgagcttcgggttggaagtgggtgggagagttcgaggcct
tgcgcttaaggagccccttcgcctcgtgcttgagttgaggcctggcctgggcgctggggccgccgcgtgcgaatctggtggcaccttcgcgcctgtctcgctgct
ttcgataagtctctagccatttaaaatttttgatgacctgctgcgacgctttttttctggcaagatagtcttgtaaatgcgggccaagatctgcacactggtatttcggtttt
tggggccgcgggcggcgacggggcccgtgcgtcccagcgcacatgttcggcgaggcggggcctgcgagcgcggccaccgagaatcggacgggggtagt
ctcaagctggccggcctgctctggtgcctggcctcgcgccgccgtgtatcgccccgccctgggcggcaaggctggcccggtcggcaccagttgcgtgagcg
gaaagatggccgcttcccggccctgctgcagggagctcaaaatggaggacgcggcgctcgggagagcgggcgggtgagtcacccacacaaaggaaaagg
gcctttccgtcctcagccgtcgcttcatgtgactccacggagtaccgggcgccgtccaggcacctcgattagttctcgagcttttggagtacgtcgtctttaggttgg
ggggaggggttttatgcgatggagtttccccacactgagtgggtggagactgaagttaggccagcttggcacttgatgtaattctccttggaatttgccctttttgagt
ttggatcttggttcattctcaagcctcagacagtggttcaaagtttttttcttccatttcaggtgtcgtgacgtacggccaccatgaaaaggccggcggccacgaaaa
aggccggccaggcaaaaaagaaaaaggacaagaagtacagcatcggcctggccatcggcaccaactctgtgggctgggccgtgatcaccgacgagtacaa
ggtgcccagcaagaaattcaaggtgctgggcaacaccgaccggcacagcatcaagaagaacctgatcggagccctgctgttcgacagcggcgaaacagccg
aggccacccggctgaagagaaccgccagaagaagatacaccagacggaagaaccggatctgctatctgcaagagatcttcagcaacgagatggccaaggtg
gacgacagcttcttccacagactggaagagtccttcctggtggaagaggataagaagcacgagcggcaccccatcttcggcaacatcgtggacgaggtggcct
accacgagaagtaccccaccatctaccacctgagaaagaaactggtggacagcaccgacaaggccgacctgcggctgatctatctggccctggcccacatga
tcaagttccggggccacttcctgatcgagggcgacctgaaccccgacaacagcgacgtggacaagctgttcatccagctggtgcagacctacaaccagctgttc
gaggaaaaccccatcaacgccagcggcgtggacgccaaggccatcctgtctgccagactgagcaagagcagacggctggaaaatctgatcgcccagctgcc
cggcgagaagaagaatggcctgttcggcaacctgattgccctgagcctgggcctgacccccaacttcaagagcaacttcgacctggccgaggatgccaaactg
cagctgagcaaggacacctacgacgacgacctggacaacctgctggcccagatcggcgaccagtacgccgacctgtttctggccgccaagaacctgtccgac
gccatcctgctgagcgacatcctgagagtgaacaccgagatcaccaaggcccccctgagcgcctctatgatcaagagatacgacgagcaccaccaggacctg
accctgctgaaagctctcgtgcggcagcagctgcctgagaagtacaaagagattttcttcgaccagagcaagaacggctacgccggctacattgacggcggag
ccagccaggaagagttctacaagttcatcaagcccatcctggaaaagatggacggcaccgaggaactgctcgtgaagctgaacagagaggacctgctgcgga
agcagcggaccttcgacaacggcagcatccccaccagatccacctgggagagctgcacgccattctgcggcggcaggaagatttttacccattcctgaagga
caaccgggaaaagatcgagaagatcctgaccttccgcatcccctactacgtgggccctctggccaggggaaacagcagattcgcctggatgaccagaaagag
cgaggaaaccatcacccccctggaacttcgaggaagtggtggacaagggcgcttccgcccagagcttcatcgagcggatgaccaacttcgataagaacctgcc

caacgagaaggtgctgcccaagcacagcctgctgtacgagtacttcaccgtgtataacgagctgaccaaagtgaaatacgtgaccgagggaatgagaaagcc
cgccttcctgagcggcgagcagaaaaaggccatcgtggacctgctgttcaagaccaaccggaaagtgaccgtgaagcagctgaaagaggactacttcaagaa
aatcgagtgcttcgactccgtggaaatctccggcgtggaagatcggttcaacgcctccctgggcacataccacgatctgctgaaaattatcaaggacaaggactt
cctggacaatgaggaaaacgaggacattctggaagatatcgtgctgaccctgacactgtttgaggacagagagatgatcgaggaacggctgaaaacctatgcc
cacctgttcgacgacaaagtgatgaagcagctgaagcggcggagatacaccggctggggcaggctgagccggaagctgatcaacggcatccgggacaagc
agtccggcaagacaatcctggatttcctgaagtccgacggcttcgccaacagaaacttcatgcagctgatccacgacgacagcctgacctttaaagaggacatc
cagaaagcccaggtgtccggccagggcgatagcctgcacgagcacattgccaatctggccggcagccccgccattaagaagggcatcctgcagacagtgaa
ggtggtggacgagctcgtgaaagtgatgggccggcacaagcccgagaacatcgtgatcgaaatggccagagagaaccagaccacccagaagggacagaa
gaacagccgcgagagaatgaagcggatcgaagagggcatcaaagagctgggcagccagatcctgaaagaacaccccgtggaaaacacccagctgcagaa
cgagaagctgtacctgtactacctgcagaatgggcgggatatgtacgtggaccaggaactggacatcaaccggctgtccgactacgatgtggaccacatcgtg
cctcagagctttctgaaggacgactccatcgacaacaaggtgctgaccagaagcgacaaggcccggggcaagagcgacaacgtgccctccgaagaggtcgt
gaagaagatgaagaactactggcggcagctgctgaacgccaagctgattacccagagaaagttcgacaatctgaccaaggccgagagaggcggcctgagcg
aactggataaggccggcttcatcaagagacagctggtggaaacccggcagatcacaaagcacgtggcacagatcctggactcccggatgaacactaagtacg
acgagaatgacaagctgatccgggaagtgaaagtgatcaccctgaagtccaagctggtgtccgatttccggaaggatttccagttttacaaagtgcgcgagatca
acaactaccaccacgcccacgacgcctacctgaacgccgtcgtgggaaccgccctgatcaaaaagtaccctaagctggaaagcgagttcgtgtacggcgact
acaaggtgtacgacgtgcggaagatgatcgccaagagcgagcaggaaatcggcaaggctaccgccaagtacttcttctacagcaacatcatgaactttttcaag
accgagattaccctggccaacggcgagatccggaagcggcctctgatcgagacaaacggcgaaaccggggagatcgtgtgggataagggccgggattttgc
caccgtgcggaaagtgctgagcatgccccaagtgaatatcgtgaaaaagaccgaggtgcagacaggcggcttcagcaaagagtctatcctgcccaagaggaa
cagcgataagctgatcgccagaaagaaggactgggaccctaagaagtacggcggcttcgacagccccaccgtggcctattctgtgctggtggtggccaaagt
ggaaaagggcaagtccaagaaactgaagagtgtgaaagagctgctggggatcaccatcatggaaagaagcagcttcgagaagaatcccatcgactttctgga
agccaagggctacaaagaagtgaaaaaggacctgatcatcaagctgcctaagtactccctgttcgagctggaaaacggccggaagagaatgctggcctctgcc
ggcgaactgcagaagggaaacgaactggccctgccctccaaatatgtgaacttcctgtacctggccagccactatgagaagctgaagggctcccccgaggata
atgagcagaaacagctgtttgtggaacagcacaagcactacctggacgagatcatcgagcagatcagcgagttctccaagagagtgatcctggccgacgctaa
tctggacaaagtgctgtccgcctacaacaagcaccgggataagcccatcagagagcaggccgagaatatcatccacctgtttaccctgaccaatctgggagccc
ctgccgccttcaagtactttgacaccaccatcgaccggaagaggtacaccagcaccaaagaggtgctggacgccaccctgatccaccagagcatcaccggcct
gtacgagacacggatcgacctgtctcagctgggaggcgacagcgctggaggaggtggaagcggaggaggaggaagcggaggaggaggtagcggaccta
agaaaaagaggaaggtggcggccgctggatccGCAGAAATCGGTACTGGCTTTCcattcgacccccattatgtggaagtcctgggcgag
cgcatgcactacgtcgatgttggtccgcgcgatggcacccctgtgctgttcctgcacggtaacccgacctcctcctacgtgtggcgcaacatcatcccgcatgttg
caccgacccatcgctgcattgctccagacctgatcggtatgggcaaatccgacaaaccagacctgggttatttcttcgacgaccacgtccgcttcatggatgcctt
catcgaagccctgggtctggaagaggtcgtcctggtcattcacgactggggctccgctctgggtttccactgggccaagcgcaatccagagcgcgtcaaaggta
ttgcatttatggagttcatccgccctatcccgacctgggacgaatggccagaatttgcccgcgagaccttccaggccttccgcaccaccgacgtcggccgcaag
ctgatcatcgatcagaacgtttttatcgagggtacgctgccgatgggtgtcgtccgcccgctgactgaagtcgagatggaccattaccgcgagccgttcctgaatc
ctgttgaccgcgagccactgtggcgcttcccaaacgagctgccaatcgccggtgagccagcgaacatcgtcgcgctggtcgaagaatacatggactggctgca
ccagtcccctgtcccgaagctgctgttctggggcaccccaggcgttctgatcccaccggccgaagccgctcgcctggccaaaagcctgcctaactgcaaggct
gtggacatcggcccgggtctgaatctgctgcaagaagacaacccggacctgatcggcagcgagatcgcgcgctggctgTCTACTCTGGAGATT
TCCGGTTgtacagagggcagaggaagtctgctaacatgcggtgacgtggaggagaatcccggccctgctagcatggtgagcaagggcgaggaggata
acatggccatcatcaaggagttcatgcgcttcaaggtgcacatggagggctccgtgaacggccacgagttcgagatcgagggcgagggcgagggccgcccc
tacgagggcacccagaccgccaagctgaaggtgaccaagggtggccccctgcccttcgcctgggacatcctgtcccctcagttcatgtacggctccaaggcct
acgtgaagcaccccgccgacatccccgactacttgaagctgtccttccccgagggcttcaagtgggagcgcgtgatgaacttcgaggacggcggcgtggtga
ccgtgacccaggactcctccctgcaggacggcgagttcatctacaaggtgaagctgcgcggcaccaacttcccctcagacggccccgtaatgcagaagaaaa
ccatgggctgggaggcctcctccgagcggatgtaccccgaggacggcgccctgaagggcgagatcaagcagaggctgaagctgaaggacggcggccact
acgacgctgaggtcaagaccacctacaaggccaagaagcccgtgcagctgcccggcgcctacaacgtcaacatcaagttggacatcacctcccacaacgag
gactacaccatcgtggaacagtacgaacgcgccgagggccgccactccaccggcggcatggacgagctgtacaagtaagaattcgatatcaagcttatcggta
atcaacctctggattacaaaatttgtgaaagattgactggtattcttaactatgttgctccttttacgctatgtggatacgctgctttaatgcctttgtatcatgctattgcttc
ccgtatggctttcattttctcctccttgtataaatcctggttgctgtctctttatgaggagttgtggcccgttgtcaggcaacgtggcgtggtgtgcactgtgtttgctgac
gcaacccccactggttggggcattgccaccacctgtcagctcctttccgggactttcgctttccccctccctattgccacggcggaactcatcgccgcctgccttgc

ccgctgctggacaggggctcggctgttgggcactgacaattccgtggtgttgtcggggaaatcatcgtcctttccttggctgctcgcctgtgttgccacctggattct
gcgcgggacgtccttctgctacgtcccttcggccctcaatccagcggaccttccttcccgcggcctgctgccggctctgcggcctcttccgcgtcttcgccttcgc
cctcagacgagtcggatctccctttgggccgcctccccgcatcgataccgtcgacctcgagacctagaaaaacatggagcaatcacaagtagcaatacagcag
ctaccaatgctgattgtgcctggctagaagcacaagaggaggaggaggtgggttttccagtcacacctcaggtacctttaagaccaatgacttacaaggcagctg
tagatcttagccactttttaaaagaaaaggggggactggaagggctaattcactcccaacgaagacaagatatccttgatctgtggatctaccacacacaaggcta
cttccctgattggcagaactacacaccagggccagggatcagatatccactgacctttggatggtgctacaagctagtaccagttgagcaagagaaggtagaag
aagccaatgaaggagagaacacccgcttgttacaccctgtgagcctgcatgggatggatgacccggagagagaagtattagagtggaggtttgacagccgcct
agcatttcatcacatggcccgagagctgcatccggactgtactgggtctctctggttagaccagatctgagcctgggagctctctggctaactagggaacccactg
cttaagcctcaataaagcttgccttgagtgcttcaagtagtgtgtgcccgtctgttgtgtgactctggtaactagagatccctcagacccttttagtcagtgtggaaaat
ctctagcagggcccgtttaaacccgctgatcagcctcgactgtgccttctagttgccagccatctgttgtttgcccctcccccgtgccttccttgaccctggaaggtg
ccactcccactgtcctttcctaataaaatgaggaaattgcatcgcattgtctgagtaggtgtcattctattctggggggtggggtggggcaggacagcaaggggga
ggattgggaagacaatagcaggcatgctggggatgcggtgggctctatggcttctgaggcggaaagaaccagctggggctctagggggtatccccacgcgcc
ctgtagcggcgcattaagcgcggcgggtgtggtggttacgcgcagcgtgaccgctacacttgccagcgccctagcgcccgctcctttcgctttcttcccttcctttc
tcgccacgttcgccggctttccccgtcaagctctaaatcgggggctccctttagggttccgatttagtgctttacggcacctcgaccccaaaaaacttgattagggt
gatggttcacgtagtgggccatcgccctgatagacggtttttcgccctttgacgttggagtccacgttctttaatagtggactcttgttccaaactggaacaacactca
accctatctcggtctattcttttgatttataagggattttgccgatttcggcctattggttaaaaaatgagctgatttaacaaaaatttaacgcgaattaattctgtggaatg
tgtgtcagttagggtgtggaaagtccccaggctccccagcaggcagaagtatgcaaagcatgcatctcaattagtcagcaaccaggtgtggaaagtccccaggc
tccccagcaggcagaagtatgcaaagcatgcatctcaattagtcagcaaccatagtcccgcccctaactccgcccatcccgcccctaactccgcccagttccgcc
cattctccgccccatggctgactaattttttttatttatgcagaggccgaggccgcctctgcctctgagctattccagaagtagtgaggaggcttttttggaggcctag
gcttttgcaaaaagctcccgggagcttgtatatccattttcggatctgatcagcacgtgttgacaattaatcatcggcatagtatatcggcatagtataatacgacaag
gtgaggaactaaaccatggccaagttgaccagtgccgttccggtgctcaccgcgcgcgacgtcgccggagcggtcgagttctggaccgaccggctcgggttct
cccgggacttcgtggaggacgacttcgccggtgtggtccgggacgacgtgaccctgttcatcagcgcggtccaggaccaggtggtgccggacaacaccctgg
cctgggtgtgggtgcgcggcctggacgagctgtacgccgagtggtcggaggtcgtgtccacgaacttccgggacgcctccgggccggccatgaccgagatc
ggcgagcagccgtgggggcgggagttcgccctgcgcgacccggccggcaactgcgtgcacttcgtggccgaggagcaggactgacacgtgctacgagatt
tcgattccaccgccgccttctatgaaaggttgggcttcggaatcgttttccgggacgccggctggatgatcctccagcgcggggatctcatgctggagttcttcgc
ccaccccaacttgtttattgcagcttataatggttacaaataaagcaatagcatcacaaatttcacaaataaagcatttttttcactgcattctagttgtggtttgtccaaa
ctcatcaatgtatcttatcatgtctgtataccgtcgacctctagctagagcttggcgtaatcatggtcatagctgtttcctgtgtgaaattgttatccgctcacaattccac
acaacatacgagccggaagcataaagtgtaaagcctggggtgcctaatgagtgagctaactcacattaattgcgttgcgctcactgcccgctttccagtcgggaa
acctgtcgtgccagctgcattaatgaatcggccaacgcgcggggagaggcggtttgcgtattgggcgctcttccgcttcctcgctcactgactcgctgcgctcgg
tcgttcggctgcggcgagcggtatcagctcactcaaaggcggtaatacggttatccacagaatcaggggataacgcaggaaagaacatgtgagcaaaaggcc
agcaaaaggccaggaaccgtaaaaaggccgcgttgctggcgtttttccataggctccgcccccctgacgagcatcacaaaaatcgacgctcaagtcagaggtg
gcgaaacccgacaggactataaagataccaggcgtttccccctggaagctccctcgtgcgctctcctgttccgaccctgccgcttaccggatacctgtccgccttt
ctcccttcgggaagcgtggcgctttctcatagctcacgctgtaggtatctcagttcggtgtaggtcgttcgctccaagctgggctgtgtgcacgaaccccccgttca
gcccgaccgctgcgccttatccggtaactatcgtcttgagtccaacccggtaagacacgacttatcgccactggcagcagccactggtaacaggattagcagag
cgaggtatgtaggcggtgctacagagttcttgaagtggtggcctaactacggctacactagaagaacagtatttggtatctgcgctctgctgaagccagttaccttc
ggaaaaagagttggtagctcttgatccggcaaacaaaccaccgctggtagcggtggtttttttgtttgcaagcagcagattacgcgcagaaaaaaaggatctcaa
gaagatcctttgatcttttctacggggtctgacgctcagtggaacgaaaactcacgttaagggattttggtcatgagattatcaaaaaggatcttcacctagatcctttt
aaattaaaaatgaagttttaaatcaatctaaagtatatatgagtaaacttggtctgacagttaccaatgcttaatcagtgaggcacctatctcagcgatctgtctatttcg
ttcatccatagttgcctgactccccgtcgtgtagataactacgatacgggagggcttaccatctggccccagtgctgcaatgataccgcgagacccacgctcacc
ggctccagatttatcagcaataaaccagccagccggaagggccgagcgcagaagtggtcctgcaactttatccgcctccatccagtctattaattgttgccggga
agctagagtaagtagttcgccagttaatagtttgcgcaacgttgttgccattgctacaggcatcgtggtgtcacgctcgtcgtttggtatggcttcattcagctccggtt
cccaacgatcaaggcgagttacatgatcccccatgttgtgcaaaaaagcggttagctccttcggtcctccgatcgttgtcagaagtaagttggccgcagtgttatca
ctcatggttatggcagcactgcataattctcttactgtcatgccatccgtaagatgcttttctgtgactggtgagtactcaaccaagtcattctgagaatagtgtatgcg
gcgaccgagttgctcttgcccggcgtcaatacgggataataccgcgccacatagcagaactttaaaagtgctcatcattggaaaacgttcttcggggcgaaaact
ctcaaggatcttaccgctgttgagatccagttcgatgtaacccactcgtgcacccaactgatcttcagcatcttttactttcaccagcgtttctgggtgagcaaaaaca

ggaaggcaaaatgccgcaaaaaagggaataagggcgacacggaaatgttgaatactcatactcttcctttttcaatattattgaagcatttatcagggttattgtctca
tgagcggatacatatttgaatgtatttagaaaaataaacaaataggggttccgcgcacatttccccgaaaagtgccacctgac

Supplementary-6 NMR Spectra

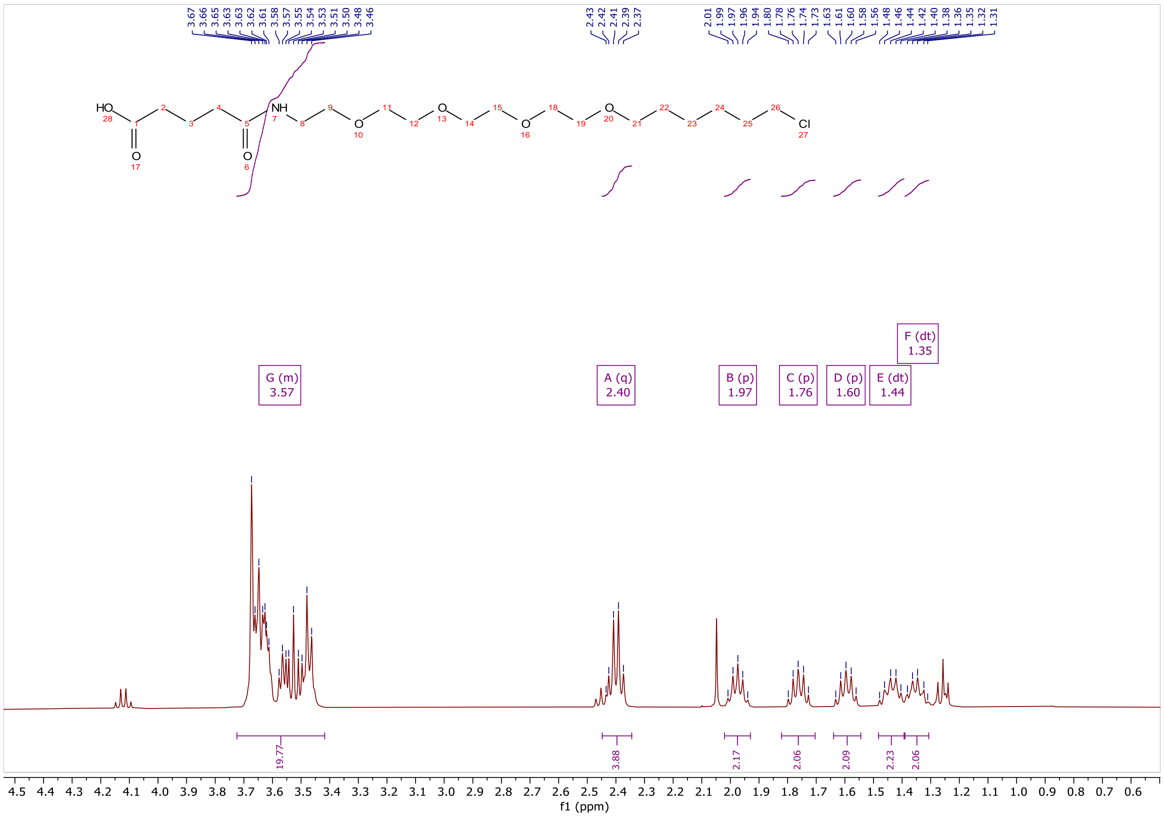

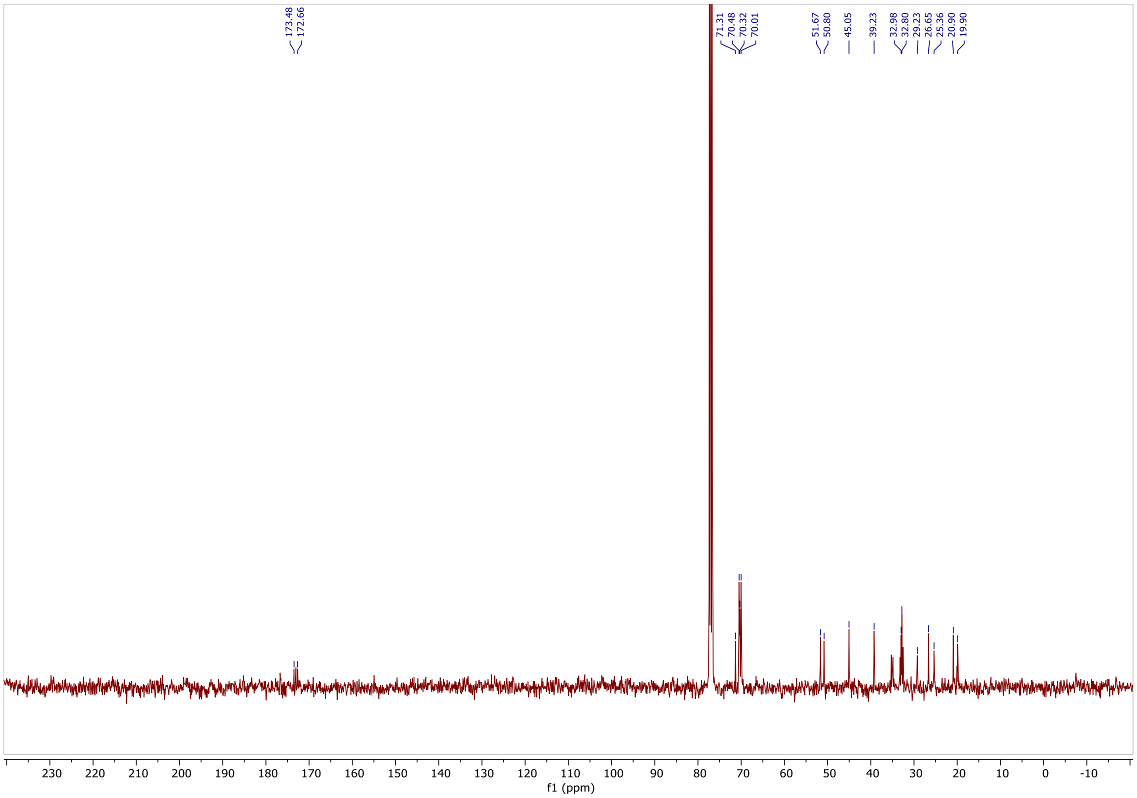

Supplementary Figure 14. ^{1}H-NMR (top) and ^{13}C-NMR spectra (bottom) of compound **Cl-PEG4-COOH**.

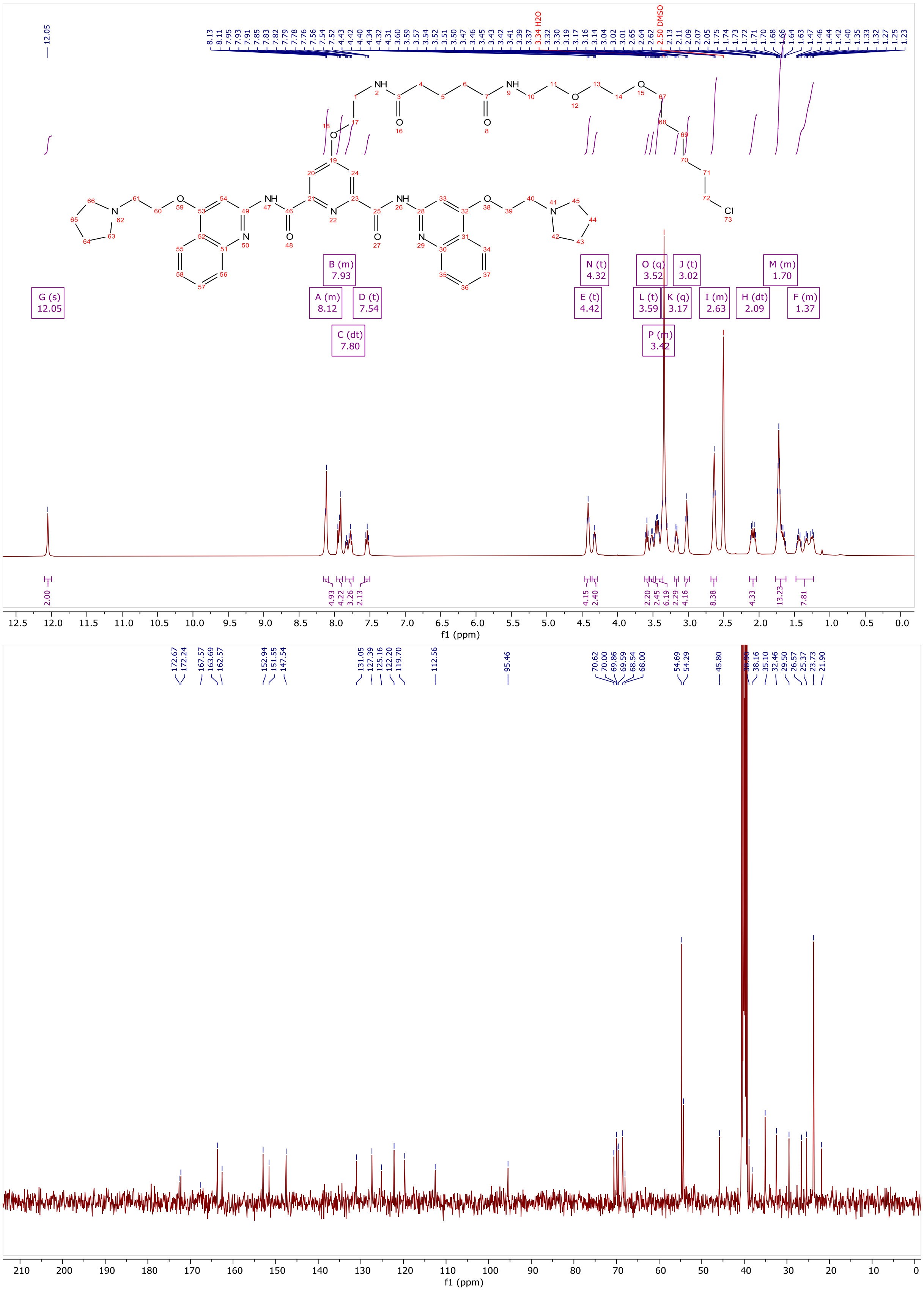

Supplementary Figure 15. ^{1}H-NMR (top) and ^{13}C-NMR spectra (bottom) of compound **Cl-PyPDS$_2$**.

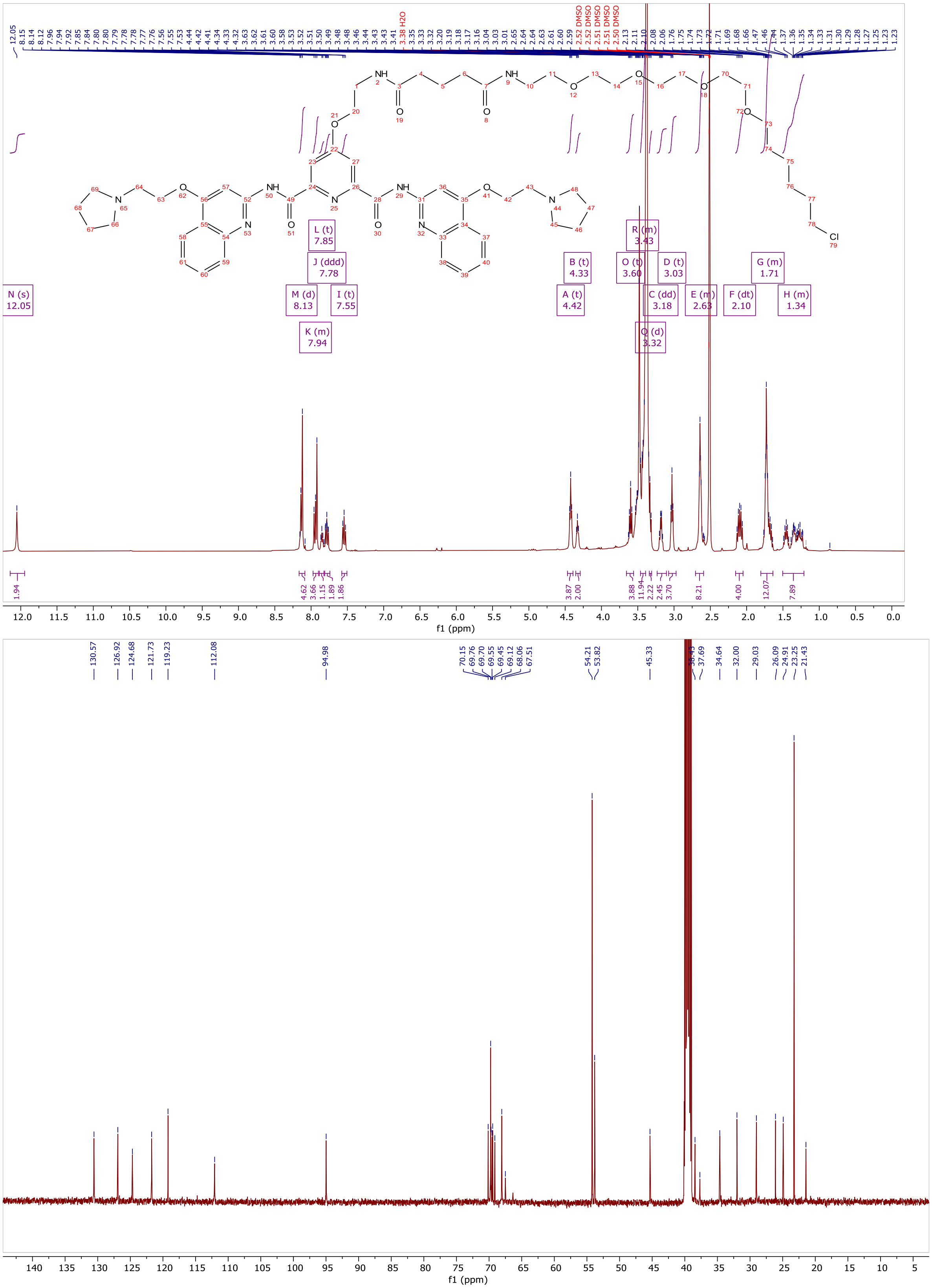

Supplementary Figure 16. ^{1}H-NMR (top) and ^{13}C-NMR spectra (bottom) of compound **Cl-PyPDS$_4$**.

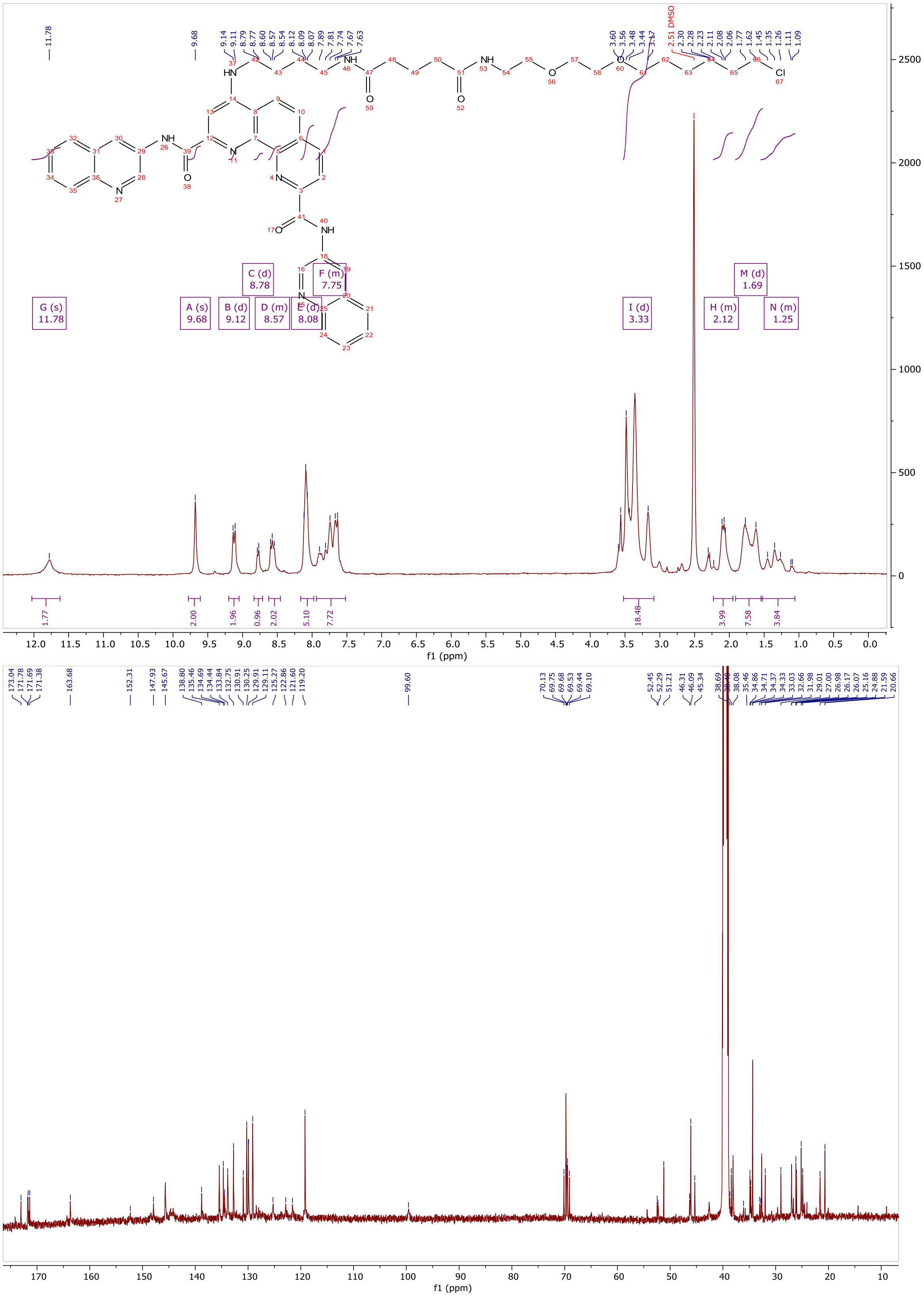

Supplementary Figure 17. ^{1}H-NMR (top) and ^{13}C-NMR spectra (bottom) of compound **S1-Cl-PhenDC3$_2$**.

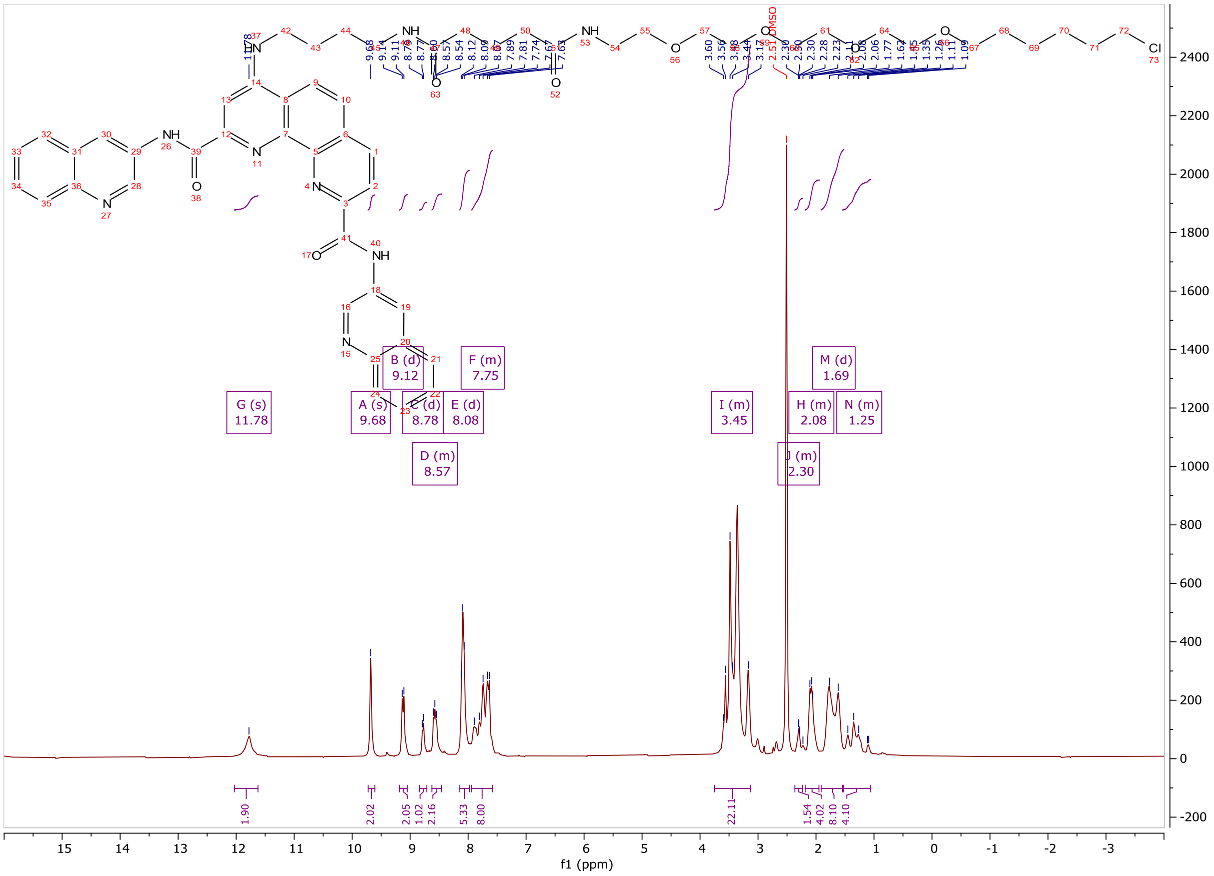

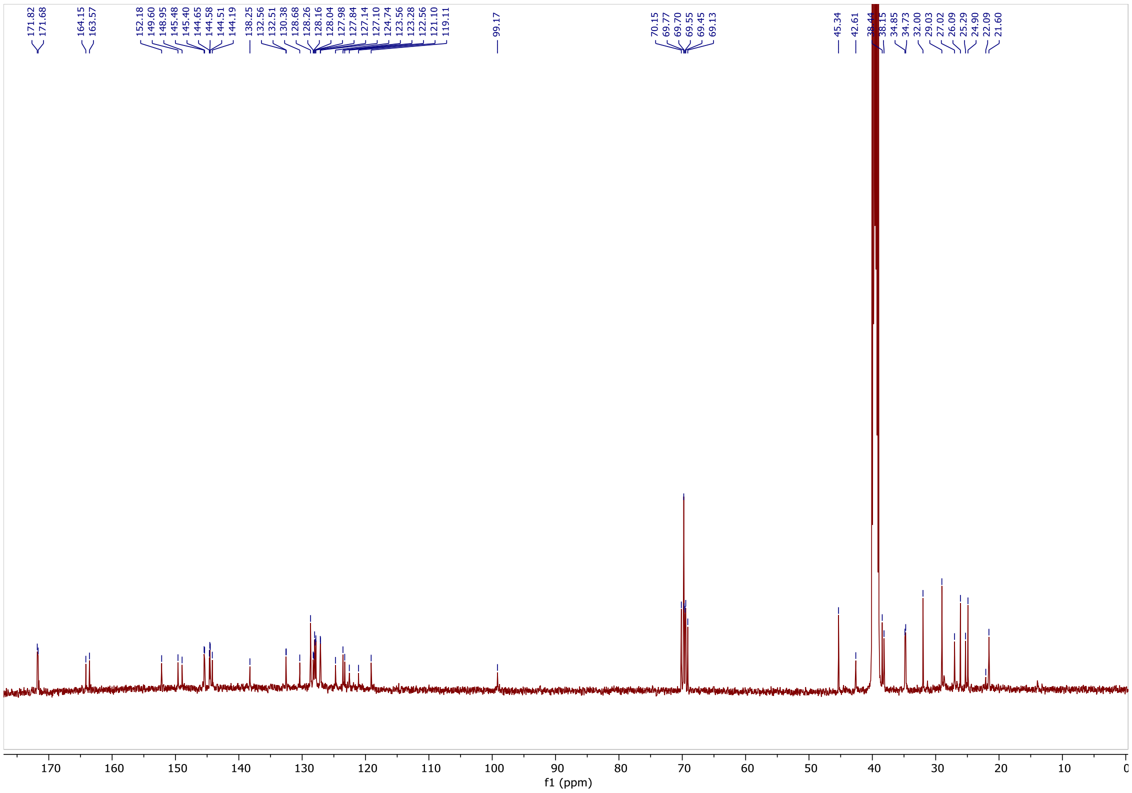

Supplementary Figure 18. ^{1}H-NMR (top) and ^{13}C-NMR spectra (bottom) of compound **S2-Cl-PhenDC3$_4$**.

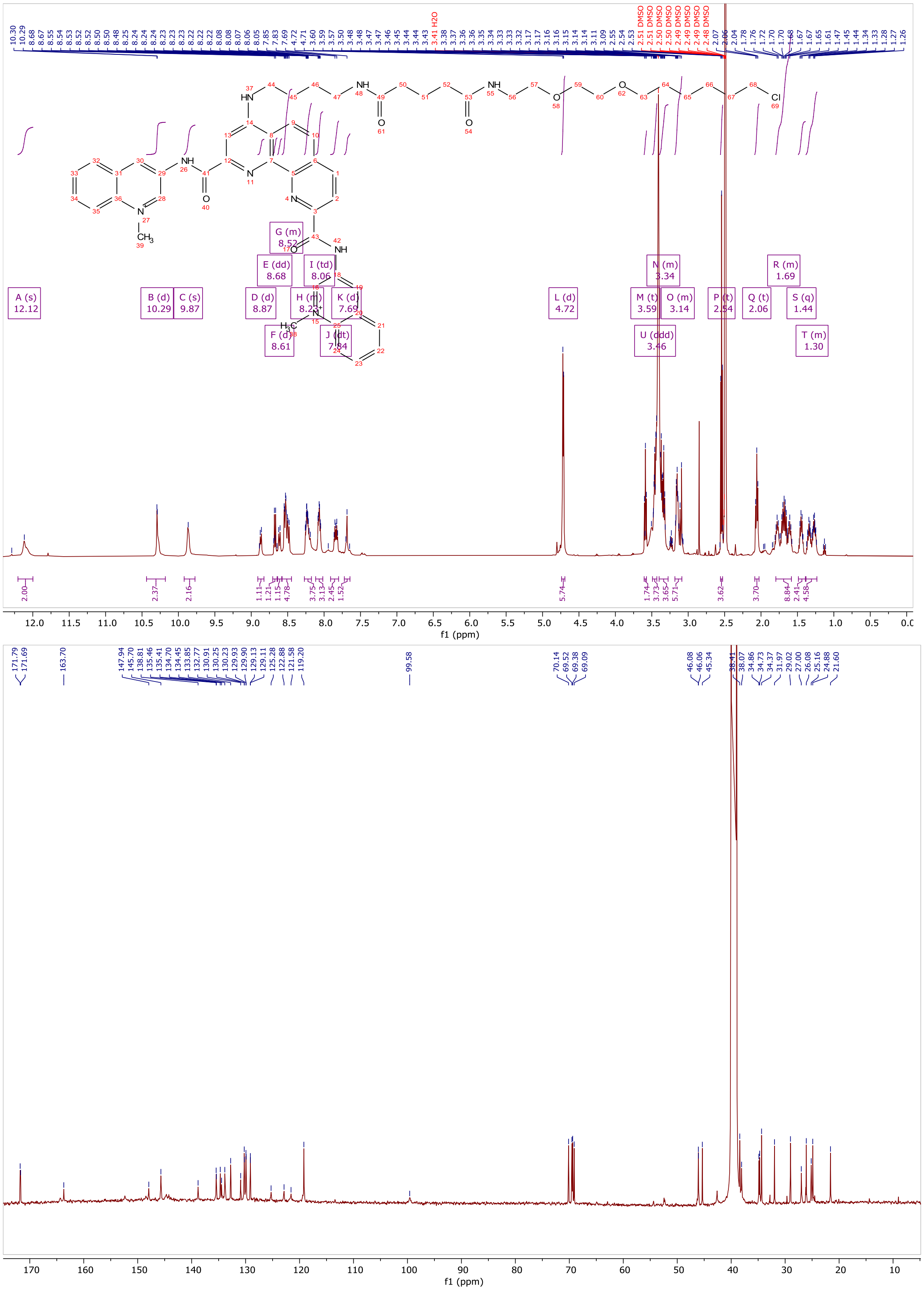

Supplementary Figure 19. ^{1}H-NMR (top) and ^{13}C-NMR spectra (bottom) of compound **Cl-PhenDC3$_2$**.

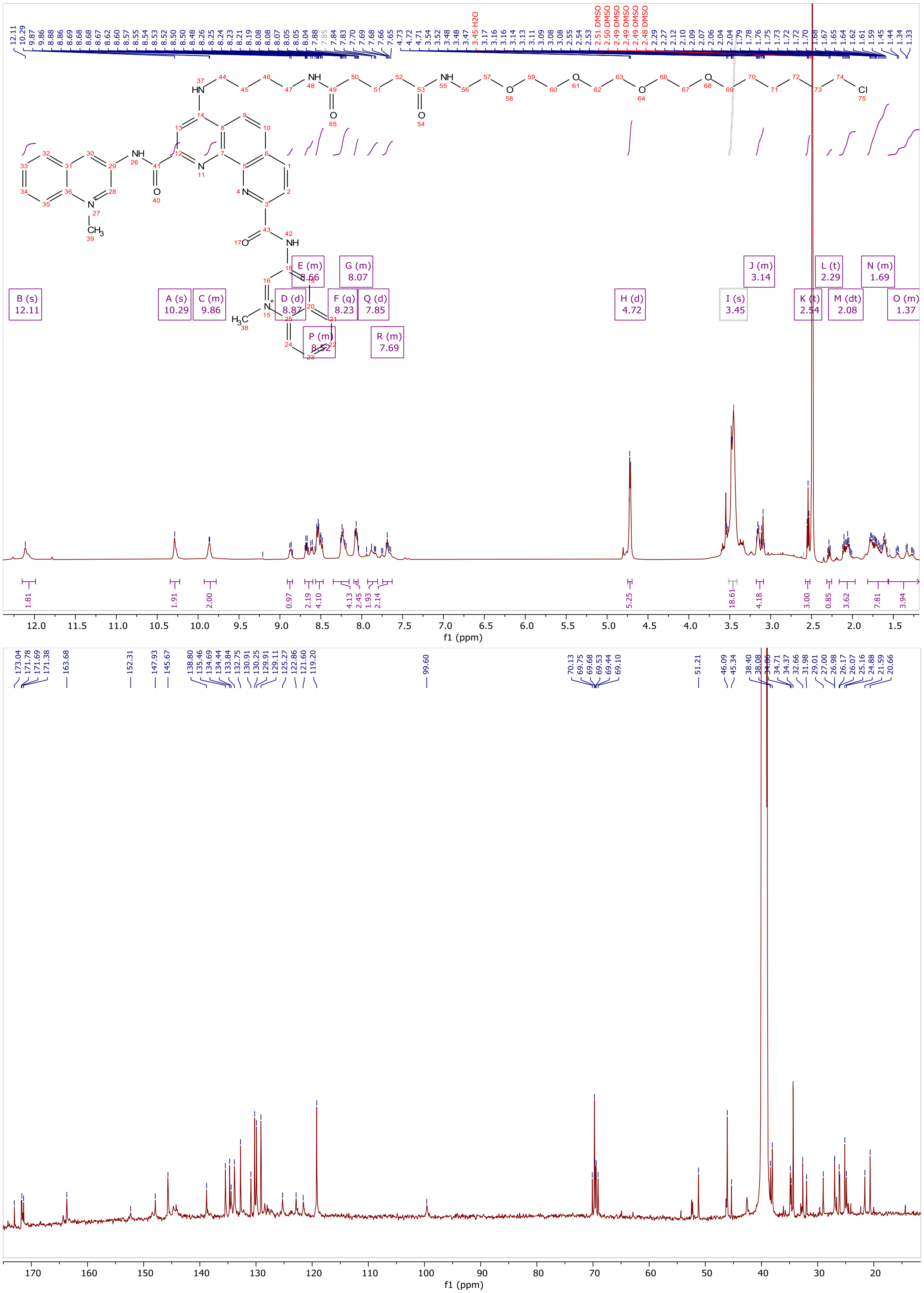

Supplementary Figure 20. ^{1}H-NMR (top) and ^{13}C-NMR spectra (bottom) of compound **Cl-PhenDC3$_4$**.

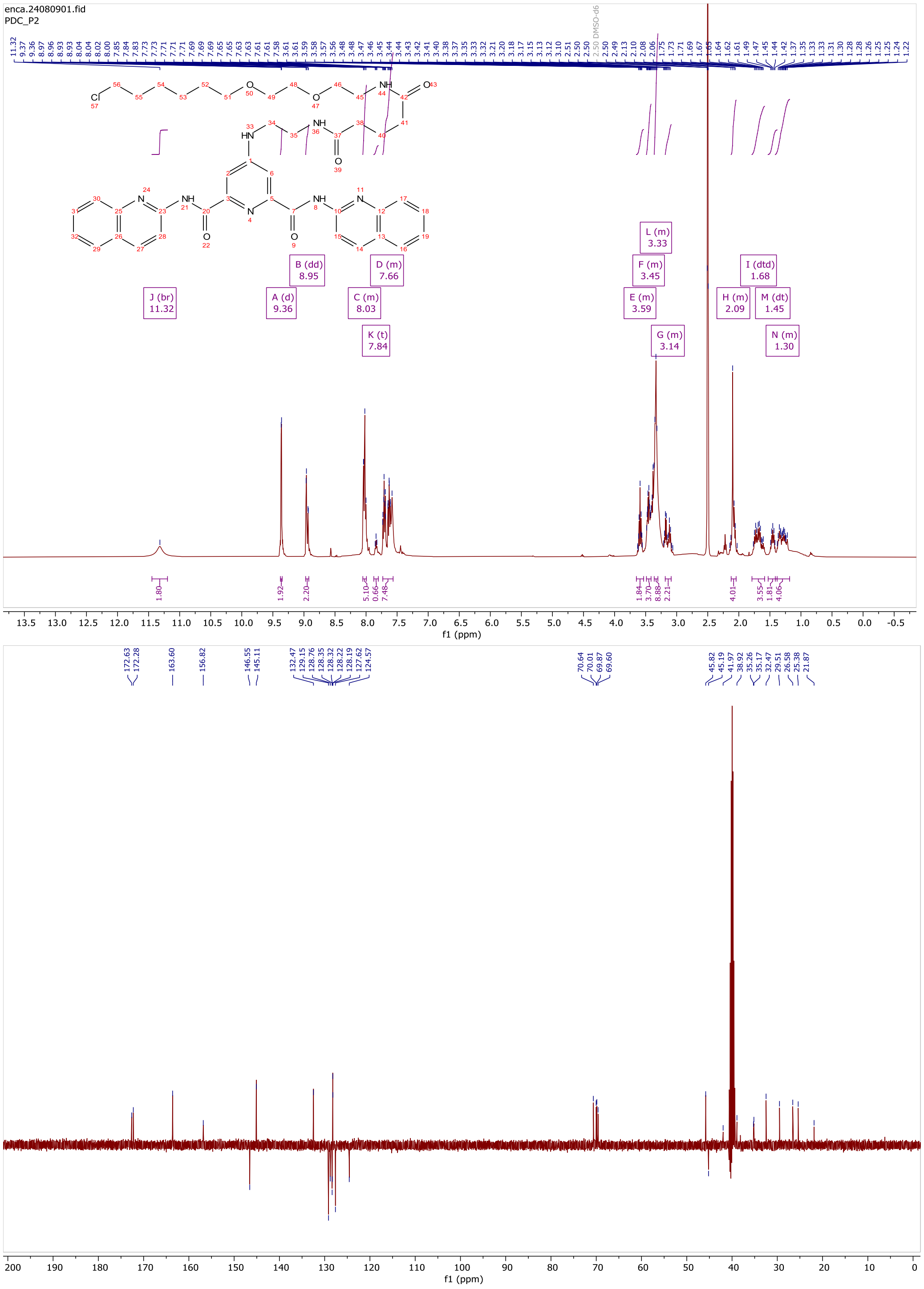

Supplementary Figure 21. ^{1}H-NMR (top) and ^{13}C-NMR spectra (bottom) of compound **S3_Cl-PDC$_2$**.

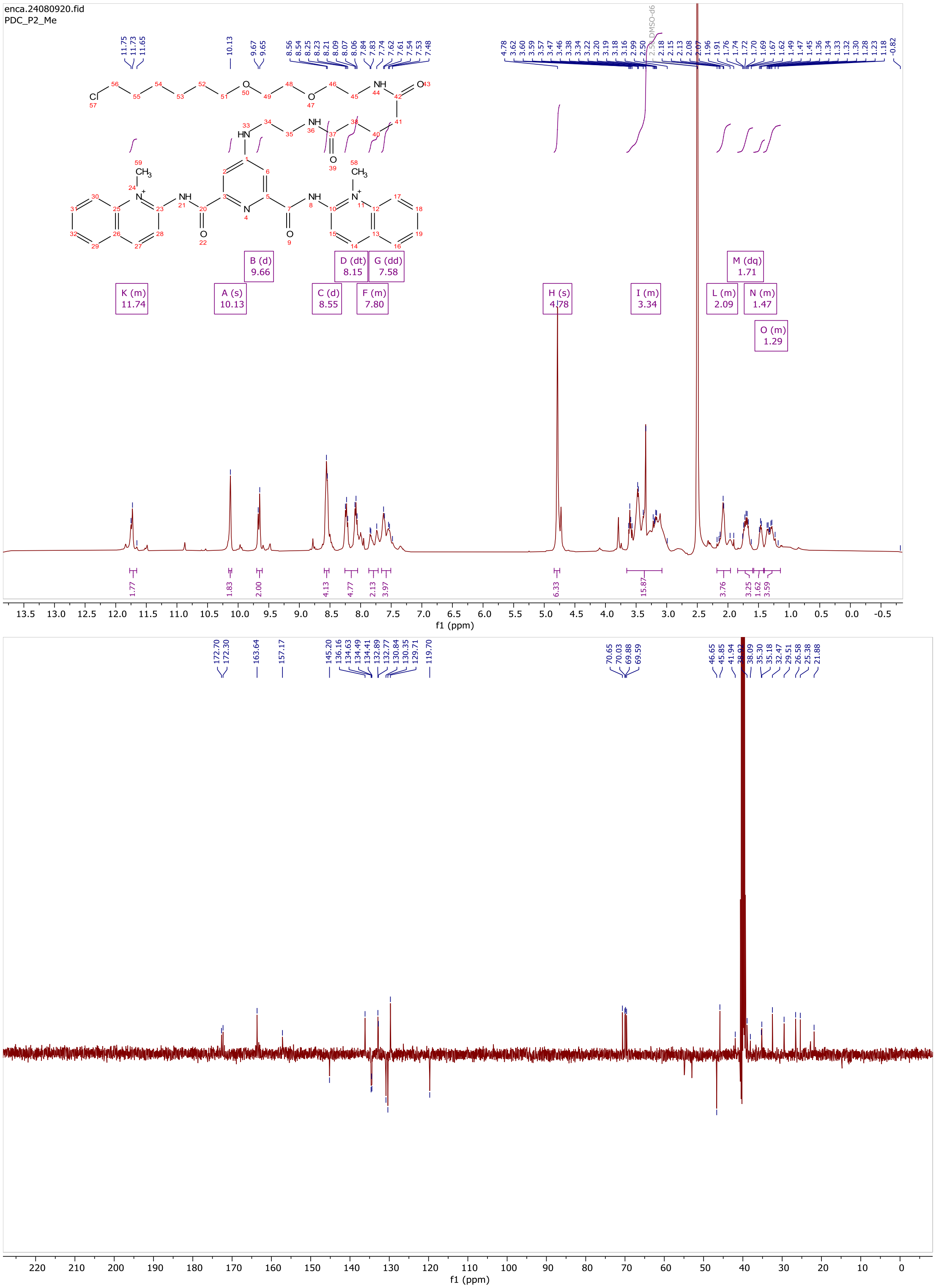

Supplementary Figure 22. ^{1}H-NMR (top) and ^{13}C-NMR spectra (bottom) of compound **Cl-PDC$_2$**.

Supplementary-7 Peptides LC-MS characterization

pep-RVS

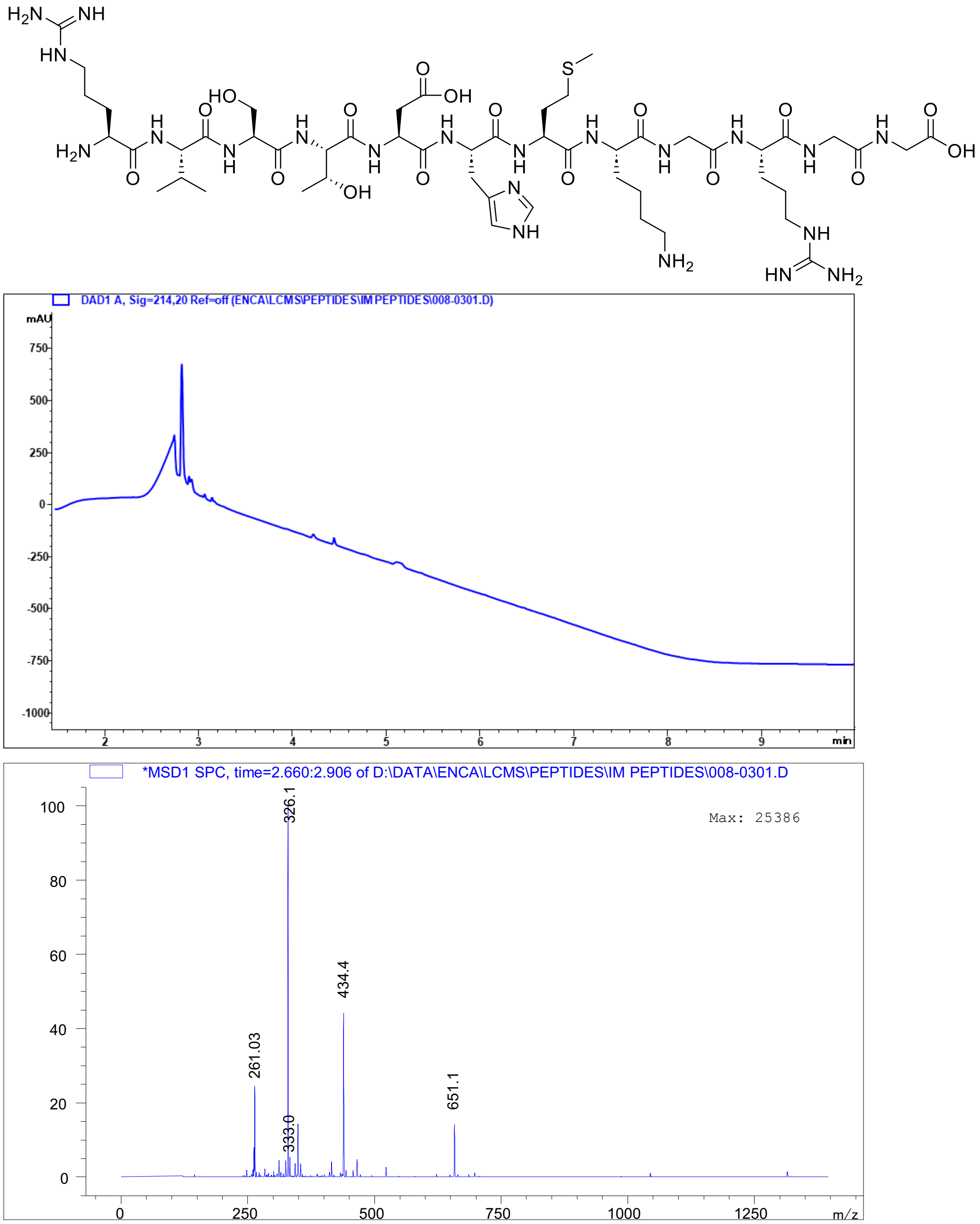

Supplementary Figure 23. HPLC-MS chromatogram of purified **IM-OH**. HPLC-UV trace at 214 nm (top) and MS spectrum of the corresponding peak (bottom). Calcd MW: 1300.4. Found: 651.1 $[M+2H]^{2+}$, 434.4 $[M+3H]^{3+}$, 326.1 $[M+4H]^{4+}$, 261.0 $[M+5H]^{5+}$.

Cl-pep-RVS$_2$

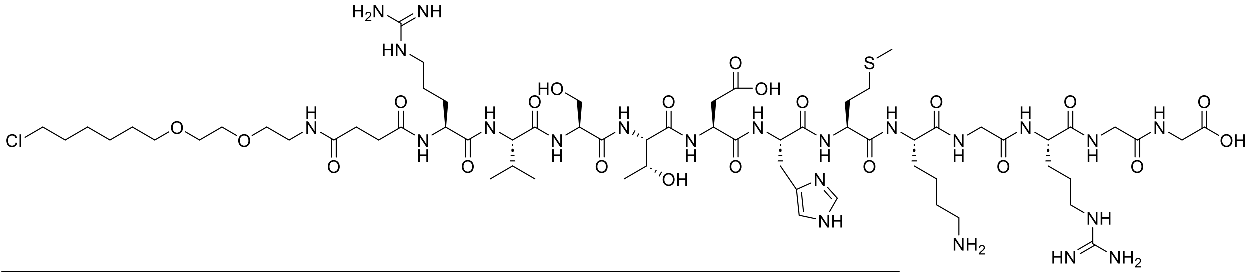

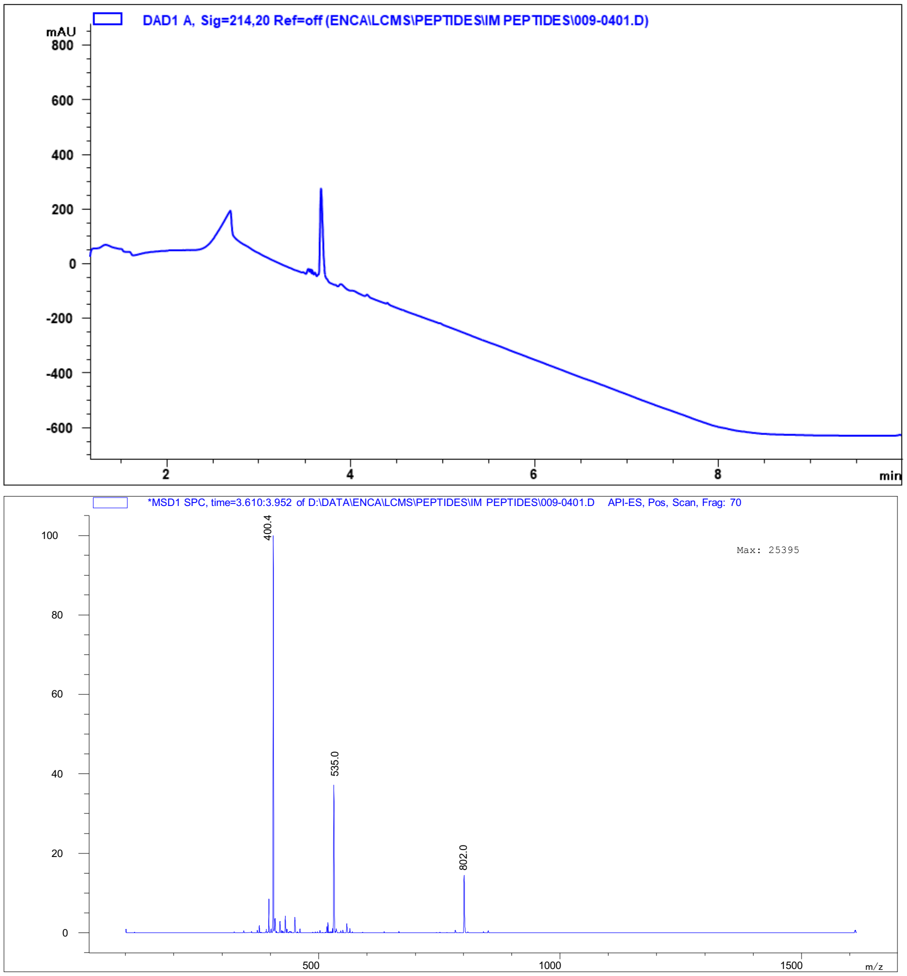

Supplementary Figure 24. HPLC-MS chromatogram of purified **IM-PEG2-CA-OH**. HPLC-UV trace at 214 nm (top) and MS spectrum of the corresponding peak (bottom). Calcd MW: 1606.2. Found: 802.0 $[M+2H]^{2+}$, 535.0 $[M+3H]^{3+}$, 400.4 $[M+4H]^{4+}$.

Cl-pep-RVS$_4$

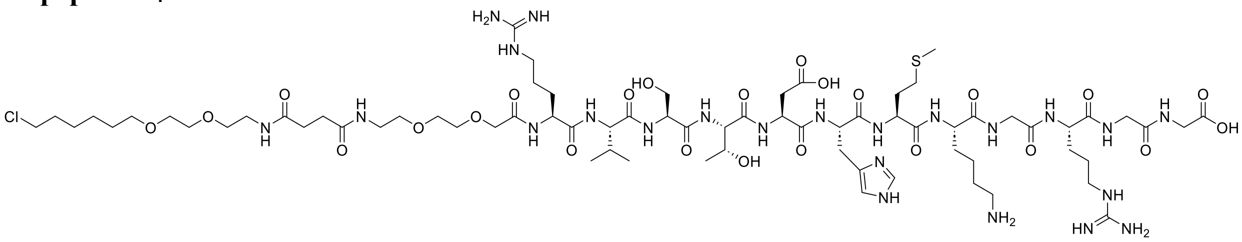

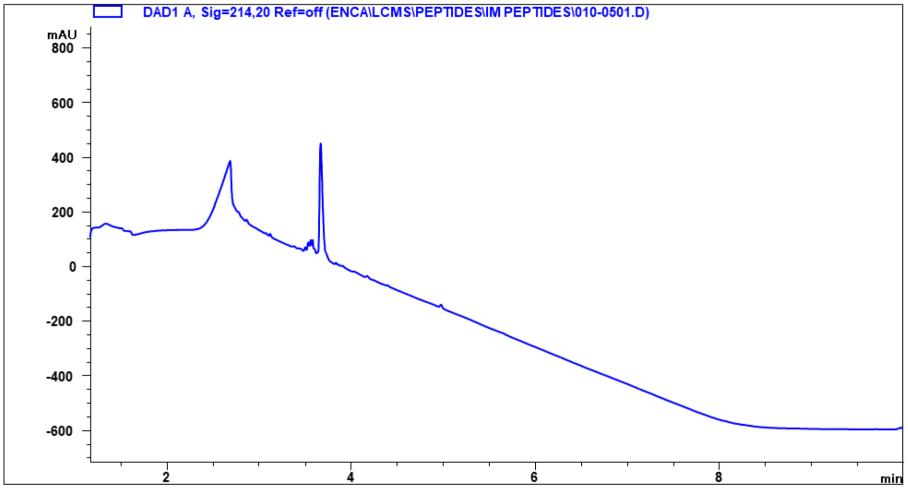

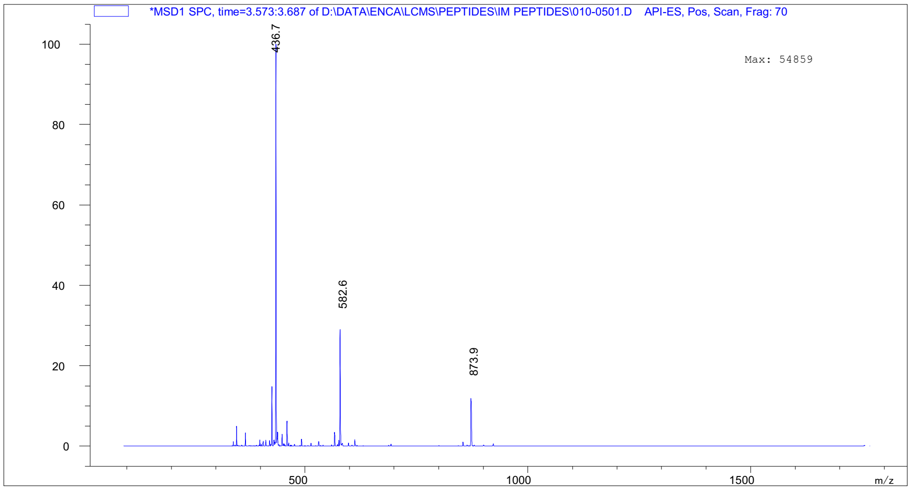

Supplementary Figure 25. HPLC-MS chromatogram of purified **IM-PEG4-CA-OH**. HPLC-UV trace at 214 nm (top) and MS spectrum of the corresponding peak (bottom). Calcd MW: 1751.4. Found: 873.9 $[M+2H]^{2+}$, 592.6 $[M+3H]^{3+}$, 436.7 $[M+4H]^{4+}$.

Supplementary-8 Cl-pep-RVS$_n$ i-motif binding characterization *via* UV-melting

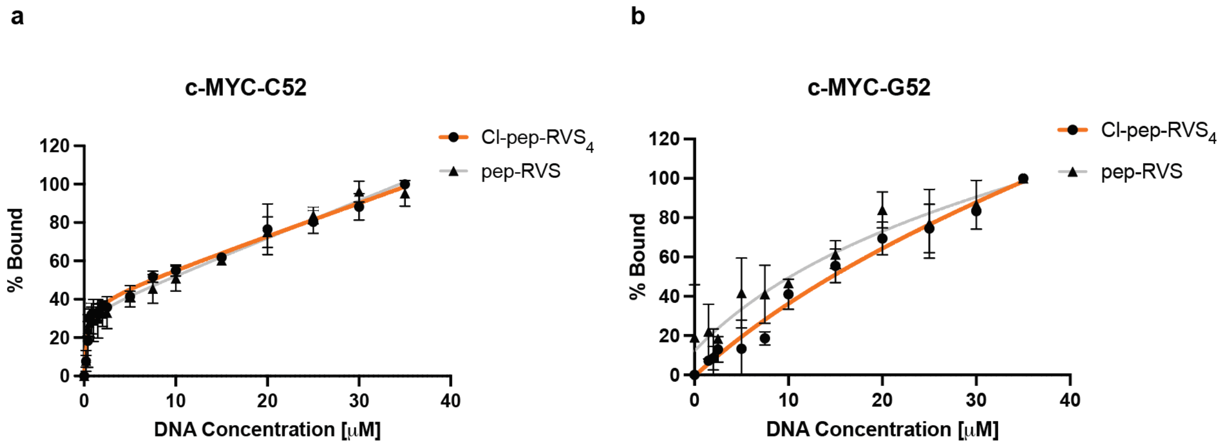

Peptide	K_d for c-MYC-C52 (Mean ± SD)
Cl-pep-RVS$_4$	0.349 ± 0.12
pep-RVS	0.250 ± 0.12

Peptide	K_d for c-MYC-G52 (Mean ± SD)
Cl-pep-RVS$_4$	32.9 ± 13.3
pep-RVS	23.6 ± 12.6

Supplementary Figure 26 UV-binding assay of Cl-pep-RVS$_n$ and pep-RVS to *c-MYC* i-motif. a, b, Normalised fraction bound (percentage) for Cl-pep-RVS$_4$ and pep-RVS interacting with c-MYC-C52 and c-MYC-G52, respectively, as a function of peptide concentration. To calculate the apparent dissociation constant (K_d), each UV-difference spectrum acquired was integrated between 325 nm and 375 nm (50 nm window) by multiplying every absorbance datum by its wavelength and summing the products. The resulting integrals were plotted against concentration, normalised to the maximal signal, and fitted with a one-site total-binding non-linear regression to extract K_d values. Error bars represent the SD of two independent titrations.

Supplementary References

1.Di Antonio, M. *et al.* Single-molecule visualisation of DNA G-quadruplex formation in live cells. *Nat. Chem.* **12**, 832–837 (2020).

2.Lefebvre, J., Guetta, C., Poyer, F., Mahuteau-Betzer, F. & Teulade-Fichou, M.-P. Copper–Alkyne Complexation Responsible for the Nucleolar Localization of Quadruplex Nucleic Acid Drugs Labeled by Click Reactions. *Angew. Chem. Int. Ed.* **56**, 11365–11369 (2017).

3.Cadoni, E. *et al.* Teaching photosensitizers a new trick: red light-triggered G-quadruplex alkylation by ligand co-localization. *Chem. Commun.* **57**, 1010–1013 (2021).

4.Nakamura, A. *et al.* Engineering Orthogonal, Plasma Membrane-Specific SLIPT Systems for Multiplexed Chemical Control of Signaling Pathways in Living Single Cells. *ACS Chem. Biol.* **15**, 1004–1015 (2020).

5. Rosano, D. et al. Long-term Multimodal Recording Reveals Epigenetic Adaptation Routes in Dormant Breast Cancer Cells. *Cancer Discov.* 14, 866-889 (2024).
