# Supplementary material for: Chemically modified CRISPR-Cas9 enables targeting of individual G-quadruplex and i-motif structures, revealing ligand-dependent transcriptional perturbation
Source: Nat Commun. 2025 Dec 9;17:385. doi: 10.1038/s41467-025-67074-z (PMC12796454; doi:10.1038/s41467-025-67074-z)
Supplement: Supplementary file 2 — Description of Additional Supplementary Files [file 41467_2025_67074_MOESM2_ESM.pdf]

Description of additional Supplementary Files

File name: Supplementary data 1

Description: Shown are normalized bigWig signal tracks mapped to the T2T-CHM13 v2.0 human reference genome. Tracks include H3K4me3 and H3K27me3 CUT&Tag histone modification profiles, as well as ATAC-seq accessibility data across the *HMGN1* locus. All CUT&Tag datasets were processed with duplicate removal and normalized using the CUTANA® k-MET spike-in. All ATAC-seq datasets were generated from duplicate-filtered, blacklist-removed, Tn5-shifted paired-end reads; fragments were restricted to properly paired reads <1000 bp, and genome-wide coverage was computed using bedtools genomecov and converted to bigWig format.

Track name: POT_k4me3_normalised_rmDup_HMGN1.bw

Description: H3K4me3 CUT&Tag normalised signal from batch CT_022, replicate B, day 0 (untreated baseline, POT).

Track name: Dormancy_k4me3_normalised_rmDup_HMGN1.bw

Description: H3K4me3 signal for batch CT_018, replicate A, Day 43 under continuous oestrogen deprivation (Dormant).

Track name: Awakening_k4me3_normalised_rmDup_HMGN1.bw

Description: H3K4me3 signal for batch CT_024, replicate A, awakening gamma.

Track name: TEP_k4me3_normalised_rmDup_HMGN1.bw

Description: H3K4me3 signal for batch CT_016, replicate A, awakening gamma expanded (Terminal End Point, TEP).

Track name: POT_k27me3_normalised_rmDup_HMGN1.bw

Description: H3K27me3 CUT&Tag normalised signal from batch CT_022, replicate B, day 0 (untreated baseline, POT)

Track name: Dormancy_k27me3_normalised_rmDup_HMGN1.bw

Description: H3K27me3 CUT&Tag signal from batch CT_018, replicate A, Day 43 under continuous oestrogen deprivation (Dormant).

Track name: Awakening_k27me3_normalised_rmDup_HMGN1.bw

Description: H3K27me3 signal for batch CT_024, replicate B, awakening gamma.

Track name: TEP_k27me3_normalised_rmDup_HMGN1.bw

Description: H3K27me3 signal for batch CT_016, replicate A, awakening gamma expanded (Terminal End Point, TEP).

Track name: POT_MCF7_ATAC_HMGN1.bw

Description: ATAC-seq accessibility profile from MCF7 cells untreated baseline (POT), replicate B

Track name: Dormancy_MCF7_ATAC_HMGN1.bw

Description: ATAC-seq signal from MCF7 cells at Dormancy (Day 43 of continuous oestrogen deprivation), replicate C.

Track name: TEP_MCF7_ATAC_HMGN1.bw

Description: ATAC-seq signal from MCF7 cells at Terminal End Point (expanded awakening), replicate gamma.
