# Supplementary material for: Chemically modified CRISPR-Cas9 enables targeting of individual G-quadruplex and i-motif structures, revealing ligand-dependent transcriptional perturbation
Source: Nat Commun. 2025 Dec 9;17:385. doi: 10.1038/s41467-025-67074-z (PMC12796454; doi:10.1038/s41467-025-67074-z)
Supplement: Supplementary file 4 — Reporting Summary [file 41467_2025_67074_MOESM4_ESM.pdf]

nature portfolio

Corresponding author(s): Marco Di Antonio

Last updated by author(s): 03/11/2025

Reporting Summary

Nature Portfolio wishes to improve the reproducibility of the work that we publish. This form provides structure for consistency and transparency in reporting. For further information on Nature Portfolio policies, see our Editorial Policies and the Editorial Policy Checklist.

Statistics

For all statistical analyses, confirm that the following items are present in the figure legend, table legend, main text, or Methods section.

n/a	Confirmed	
☐	☒	The exact sample size (*n*) for each experimental group/condition, given as a discrete number and unit of measurement
☐	☒	A statement on whether measurements were taken from distinct samples or whether the same sample was measured repeatedly
☐	☒	The statistical test(s) used AND whether they are one- or two-sided<br>*Only common tests should be described solely by name; describe more complex techniques in the Methods section.*
☒	☐	A description of all covariates tested
☒	☐	A description of any assumptions or corrections, such as tests of normality and adjustment for multiple comparisons
☐	☒	A full description of the statistical parameters including central tendency (e.g. means) or other basic estimates (e.g. regression coefficient) AND variation (e.g. standard deviation) or associated estimates of uncertainty (e.g. confidence intervals)
☐	☒	For null hypothesis testing, the test statistic (e.g. *F*, *t*, *r*) with confidence intervals, effect sizes, degrees of freedom and *P* value noted<br>*Give P values as exact values whenever suitable.*
☒	☐	For Bayesian analysis, information on the choice of priors and Markov chain Monte Carlo settings
☒	☐	For hierarchical and complex designs, identification of the appropriate level for tests and full reporting of outcomes
☐	☒	Estimates of effect sizes (e.g. Cohen's *d*, Pearson's *r*), indicating how they were calculated

Our web collection on statistics for biologists contains articles on many of the points above.

Software and code

Policy information about availability of computer code

Data collection	MxPro QPCR Software, BD FACS DIVA 9.4
Data analysis	SNAPGENE 8.0.1, GraphPad Prism Version 10.1.0 (264),

For manuscripts utilizing custom algorithms or software that are central to the research but not yet described in published literature, software must be made available to editors and reviewers. We strongly encourage code deposition in a community repository (e.g. GitHub). See the Nature Portfolio guidelines for submitting code & software for further information.

Data

Policy information about availability of data

All manuscripts must include a data availability statement. This statement should provide the following information, where applicable:

- Accession codes, unique identifiers, or web links for publicly available datasets
- A description of any restrictions on data availability
- For clinical datasets or third party data, please ensure that the statement adheres to our policy

All new datasets have been deposited in the gene expression omnibus (GEO) and can be accessible: GSE279769.

Research involving human participants, their data, or biological material

Policy information about studies with human participants or human data. See also policy information about sex, gender (identity/presentation), and sexual orientation and race, ethnicity and racism.

Reporting on sex and gender	N/A
Reporting on race, ethnicity, or other socially relevant groupings	N/A
Population characteristics	N/A
Recruitment	N/A
Ethics oversight	N/A

Note that full information on the approval of the study protocol must also be provided in the manuscript.

Field-specific reporting

Please select the one below that is the best fit for your research. If you are not sure, read the appropriate sections before making your selection.

[x] Life sciences [ ] Behavioural & social sciences [ ] Ecological, evolutionary & environmental sciences

For a reference copy of the document with all sections, see nature.com/documents/nr-reporting-summary-flat.pdf

Life sciences study design

All studies must disclose on these points even when the disclosure is negative.

Sample size	Sample size was determined as n=x following previously published standard procedure and followed by statistical analysis.
Data exclusions	n/a
Replication	Biological replicates are stated as n=x and where possible data collection included technical replicates
Randomization	n/a
Blinding	n/a

Reporting for specific materials, systems and methods

We require information from authors about some types of materials, experimental systems and methods used in many studies. Here, indicate whether each material, system or method listed is relevant to your study. If you are not sure if a list item applies to your research, read the appropriate section before selecting a response.

Materials & experimental systems

n/a	Involved in the study	
[ ]	[x]	Antibodies
[ ]	[x]	Eukaryotic cell lines
[x]	[ ]	Palaeontology and archaeology
[x]	[ ]	Animals and other organisms
[x]	[ ]	Clinical data
[x]	[ ]	Dual use research of concern
[x]	[ ]	Plants

Methods

n/a	Involved in the study	
[x]	[ ]	ChIP-seq
[ ]	[x]	Flow cytometry
[x]	[ ]	MRI-based neuroimaging

Antibodies

Antibodies used	Cas9 antibody- 14697T, Cell Signaling Technology. GAPDH antibody- 2118T, Cell Signaling Technology. Goat anti-rabbit/mouse HRP, R-05071-500, R-05072-500, Advansta. BG4, in house expressed from plasmid Addgene #55756. Tri-Methyl-Histone H3 (Lys27) ,9733S, Cell Signaling Technology. DYKDDDDK Tag Antibody, 2368S, Cell Signaling Technology. Anti-rabbit, ABIN101961, Abcam.
Validation	Cell signaling antibody: they were validated by the manufacturer using standard western blot procedures. BG4 expressed in house was in house validated using ELISA.

Eukaryotic cell lines

Policy information about cell lines and Sex and Gender in Research

Cell line source(s)	Cells were a kind gift of Luca Magnani's lab
Authentication	They were authenticated via WGS and karyotype
Mycoplasma contamination	All the cell line used in this work were tested negative for mycoplasma.
Commonly misidentified lines (See ICLAC register)	N/A

Plants

Seed stocks	N/A
Novel plant genotypes	N/A
Authentication	N/A

Flow Cytometry

Plots

Confirm that:

- [x] The axis labels state the marker and fluorochrome used (e.g. CD4-FITC).
- [x] The axis scales are clearly visible. Include numbers along axes only for bottom left plot of group (a 'group' is an analysis of identical markers).
- [ ] All plots are contour plots with outliers or pseudocolor plots.
- [x] A numerical value for number of cells or percentage (with statistics) is provided.

Methodology

Sample preparation	Cells were washed with DPBS, centrifuge (5 minutes 300 xg) and resuspended in FACS buffer (5%BSA in DPBS)
Instrument	BD FACS Aria III ,Attune NxT Flow Cytometer
Software	BD FACS DIVA 9.4, FlowJo
Cell population abundance	Clones were grown and genotyping and western blot was performed afterwards to assess the correct population.
Gating strategy	Gates were determined by analysing a control population not transduced. Positive samples in the red channel were defined as sample that contained a fluorescence >10^4

- [x] Tick this box to confirm that a figure exemplifying the gating strategy is provided in the Supplementary Information.
